# Potential neuroprotective effects of fermented foods and beverages in old age: a systematic review

**DOI:** 10.3389/fnut.2023.1170841

**Published:** 2023-06-15

**Authors:** Elena Porras-García, Irene Fernández-Espada Calderón, Juan Gavala-González, José Carlos Fernández-García

**Affiliations:** ^1^Department of Physiology, Anatomy and Cellular Biology, University of Pablo de Olavide, Seville, Spain; ^2^Department of Physical Education and Sports, University of Seville, Seville, Spain; ^3^Department of Didactics of Languages, Arts and Sport, University of Malaga, Andalucía-Tech, Instituto de Investigación Biomédica de Málaga (IBIMA), Malaga, Spain

**Keywords:** fermented-food, fermented beverages, cognitive decline, elderly, Alzheimer's disease, dementia

## Abstract

**Purpose:**

Numerous articles have recently studied the involvement of the gut microbiota in neurological diseases. Aging is associated with changes in the microbiome, which implies a reduction in microbial biodiversity among other changes. Considering that the consumption of a fermented-food diet improves intestinal permeability and barrier function, it seems of interest to study its participation in the prevention of neurodegenerative diseases. This article reviews existing studies to establish whether the consumption of fermented foods and fermented beverages prevents or ameliorates neurodegenerative decline in old age.

**Methods:**

The protocol used was performed according to the Preferred Reporting Items for Systematic Reviews and Meta-Analyses (PRISMA) guidelines. Details of the protocol for this systematic review are registered on PROSPERO (CRD42021250921).

**Results:**

Out of 465 articles identified in the Pubmed, Scopus, and Cochrane Library databases, a total of 29 that examined the relationship of the consumption of fermented products with cognitive impairment in old people were selected (22 cohort, 4 case-control, and 3 cross-sectional studies). The results suggest that low-to-moderate alcohol consumption and daily intake of coffee, soy products, and fermented-food diets in general are associated with a lower risk of dementia and Alzheimer's disease.

**Conclusion:**

Daily consumption of fermented foods and beverages, either alone or as part of a diet, has neuroprotective effects and slows cognitive decline in old people.

**Systematic review registration:**

https://www.crd.york.ac.uk/prospero/display_record.php?RecordID=250921, identifier: CRD42021250921.

## 1. Introduction

A healthy gut microbiota is responsible for synthesizing vitamins and essential amino acids and has an important role in the structural integrity of the intestinal mucosa producing neuromodulators, including bacteria-derived choline, tryptophan, intestinal-released hormones–such as ghrelin or leptin–and short-chain fatty acids (SCFA)–such as butyrate, acetate, and propionate. These metabolites are an energy and trophic-factors source for the intestinal epithelial cells, and thus strengthen the mucosal barrier (1–4), but are also crucial in the regulation of regulatory T-cell (Treg) colonies, supporting the hypothesis of their action in the brain (2, 5, 6).

Recently, the role of the gut microbiota in the central nervous system, through the microbiota gut-brain axis, has been revealed (2, 3, 5–21). Neurons in the enteric nervous system interact directly with neurochemicals produced by the gut microbiota, thus influencing signaling to the central nervous system (22).

Dysbiosis and some microbial metabolites are involved in a variety of diseases, such as inflammatory bowel disease, type 1 and type 2 diabetes mellitus, fatty liver, metabolic syndrome, obesity, cardiovascular disease, colorectal and breast cancer, hepatocellular carcinoma, asthma, osteoporosis, sarcopenia, atherosclerotic stroke, and nervous inflammatory disorders, and in neurodegenerative diseases, such as Alzheimer's disease, Parkinson's disease, vascular dementia, fibromyalgia, autism spectrum disorder, and depression (2, 4, 5, 11, 12, 14–16, 23, 24). Conditions such as age, chemicals (antibiotics, tobacco, etc.), stress, and eating habits, among others, alter the composition of the gut microbiota, producing changes at the level of the immune system. These changes are caused by increased permeability of the gut and the blood-brain barrier, leading to a chronic inflammatory response (3–6, 8, 9, 12, 15, 25–27).

It is proposed that systematic inflammatory processes may effect inflammation in the central nervous system by microglial activation, cytokines, astrocytes, neurotransmitters (serotonin, dopamine, noradrenaline, and Y-aminobutyric acid), and altered short-chain fatty acids (SCFA) secreted by bacteria, or changes in enteric neuron activity detected by the vagus nerve (3–6, 8, 12, 15, 22, 28).

Lipid accumulation in glia is a pathological characteristic of Alzheimer's disease (29). The APOE gene encodes a lipid transporter protein that serves as a ligand for membrane receptors that mediate lipoprotein uptake (30–32). The human gene encoding APOE has three isoforms that differ by two amino acids: ε3 (APOE3), ε4 (APOE4) and ε2 (APOE2) (33). The most-validated risk factor for developing the late form of Alzheimer's disease is the presence of the E4 allele of the APOE gene (APOE4) (30–32, 34). In APOE4 carriers, plaque formation is increased due to oxidation of apolipoprotein E and binding to beta-amyloid (34). APOE4 decreases the age of onset and increases the risk of developing the disease by modulating several pathways that contribute to the development of this pathogenesis, including lipid metabolism and transport (32). The existence has been shown of metabolic changes in dementia and the protective role of specific food metabolites in cognitive aging.

Considering the role of microbiome balance in neurological diseases and that numerous pathological processes require much time of performance before cognitive decline appears, it seems essential to protect its stability. There are several strategies to counteract gut dysbiosis, such as fecal microbiota transplant (35). However, diet management is an even simpler way to deal with an imbalance in the microbiota, and the consumption of fermented foods and beverages produces significant improvements in gut permeability and the barrier function (2, 4, 16, 36–38).

Fermentation is traditionally used as a biological method of food preservation. Fermented foods and beverages are defined as those prepared using microorganisms—bacteria, yeasts, and fungi- and enzymatic action to transform their components into various fermentation end-products. The type of fermentation depends on the final product. Due to their health benefits, fermented foods are considered functional foods. The process of microbial fermentation converts food substrates into nutritionally and functionally richer products, resulting in functional micro-organisms (probiotics), substrates that enhance the growth of beneficial bacteria in the gut (prebiotics) and bioactive components (biogenics). These substances act in the gastrointestinal tract by modifying the microbiota, influencing exogenous endotoxin translocation and subsequent immune activation, and promoting host nutrition (39).

When administered in adequate amounts, probiotics can exert a health benefit to the host by restoring the microbiota and maintaining immune homeostasis (40). Some probiotics, known as brain probiotics or psychobiotics, regulate neurotransmitters such as serotonin, gamma-aminobutyric acid (GABA), glutamate and brain-derived neurotrophic factor (BDNF) in learning, memory, mood, and other cognitive processes (41–43). The most common probiotic bacteria currently used are representatives of *Lactobacilli, Enterococci, Bifidobacteria*, yeasts and bacterial mixtures (44).

Prebiotics are non-digestible substrates that host microorganisms use to provide a health benefit. The three most important prebiotic compounds are the polysaccharide inulin, fructooligosaccharides (FOS) derived from various crops or sucrose, and galactooligosaccharides (GOS). These substrates are present in various fermented foods, making them symbiotic foods (with probiotic and prebiotic effect), such as cheddar, gouda and parmesan cheeses, sauerkraut (fermented cabbage), kimchi (Korean pickle made from radish and cabbage), kefir, yogurt, kombucha, tempeh (made from fermented soybeans), miso (fermented soybean paste), soy sauce and apple cider vinegar (45).

Because of the many food-microbe combinations (*Acetobacter, Leuconostoc, Lactobacillus, Streptococcus*, etc.), we find various types of fermented foods and beverages (16). In this review, we summarize the results obtained for cognitive performance in elderly individuals after the consumption of fermented products.

## 2. Materials and methods

This review was performed according to the Preferred Reporting Items for Systematic Reviews and Meta-Analyses (PRISMA) guidelines (46). Details of the protocol used are registered on PROSPERO (CRD42021250921) and can be accessed at: https://www.crd.york.ac.uk/prospero/display_record.php?RecordID=250921 (47).

### 2.1. Literature search

The articles included in this review were selected from PUBMED, SCOPUS, and Cochrane Library databases, limited to Spanish and English languages and published from 16 March 1991 to 16 June 2022. Several searches were performed in March, May, and June 2021, and in June 2022 (just before the final analysis of the results, to include possible new articles). We also include articles from manual searches.

We used Medical Subject Headings (Mesh) terms for the PUBMED search and Boolean operators for all the databases. The search followed the PICO strategy: population (Aged); intervention (Beer OR Cheese OR Koumiss OR Buttermilk OR Kefir OR Yogurt OR “Cultured milk products” OR “Soy foods” OR “Wine” OR “Fermented foods and beverages” OR “Kombucha tea” OR “Fermented beverages” OR “Fermented foods” OR “Cultured food” OR “Fermented dairy products”); and outcome (“Memory Disorders” OR “Alzheimer's disease” OR Dementia OR “Neurocognitive disorders” OR “Cognitive dysfunction” OR “Cognitive aging” OR “Mental status and dementia tests” OR “Intellectual disability” OR “Learning disabilities”).

### 2.2. Inclusion and exclusion criteria

The studies included in this review were cross-sectional, cohort, and case-control studies, which passed the Newcastle-Ottawa Scale (NOS) (48, 49).

Inclusion criteria were available full-text articles, conducted in elderly individuals (65 years or older) with preserved cognition at the initial cognitive evaluation, that investigated the relationship between consumption of fermented foods or beverages (including alcohol (wine and beer); fermented dairy products; Kombucha, fermented or semi-fermented tea; soy-based foods; and coffee and cocoa, but not supplements) and cognitive dysfunction (dementia or Alzheimer's disease) as first outcome.

Exclusion criteria applied to works done in animals or in people who were cognitively impaired at the start of the study and under 65 years old. We also excluded articles in languages other than English and Spanish, those published before 16 March 1991, and those that did not achieve 7 points or more on the Newcastle-Ottawa Scale (NOS) (48, 49).

### 2.3. Study selection and data extraction

**Figure 1:**
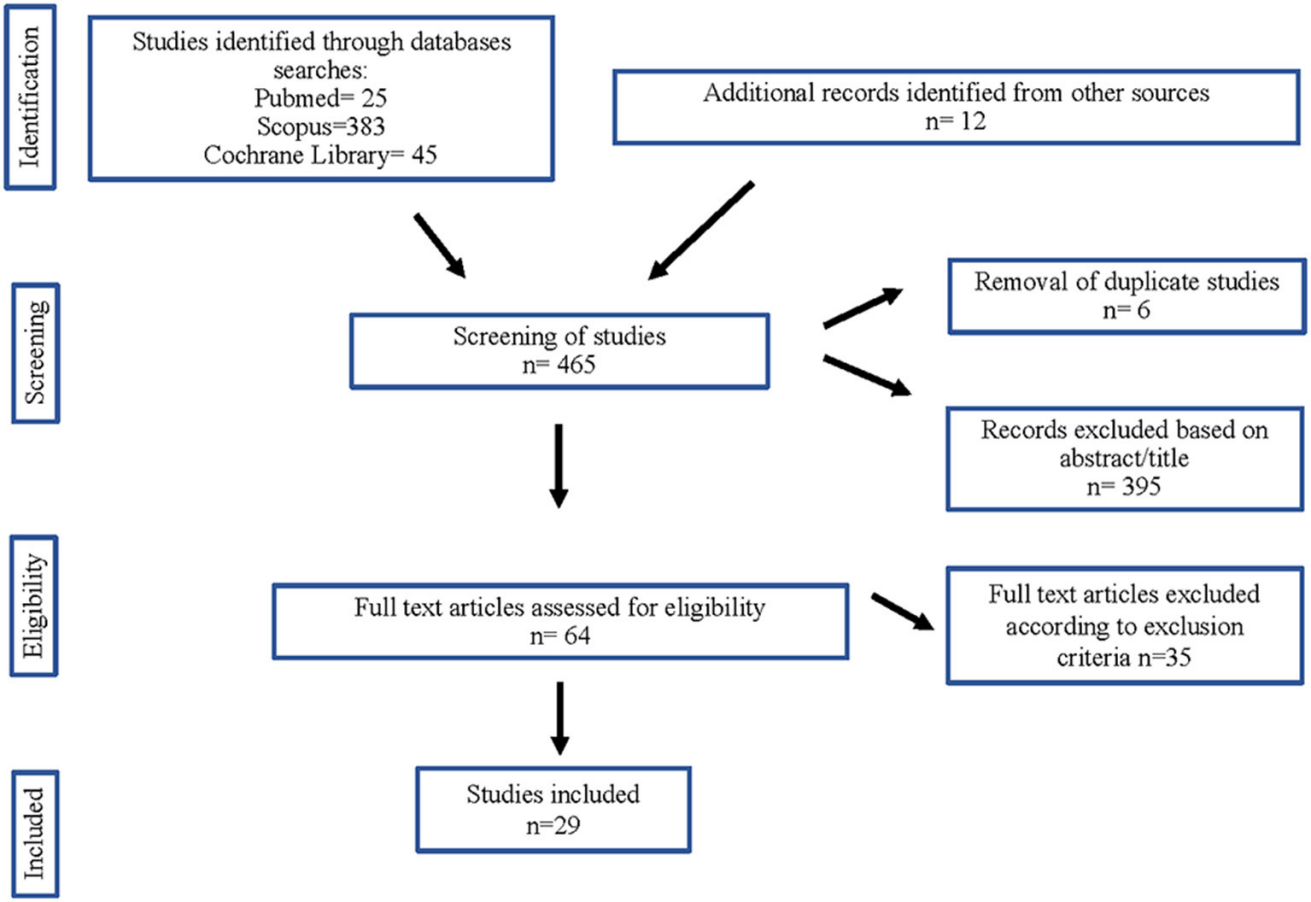
Flow diagram of studies considered for inclusion in this systematic review.

The authors independently selected and removed the duplicate studies, using Mendeley as database. The titles and the abstracts (in a first step) and the full text (in a second) of the remaining studies were screened according to the inclusion criteria. The authors extracted data from the included studies independently. The number of included and excluded studies and the selection process are illustrated in Figure 1.

From the included documents, the authors extracted the number, sex, and age of participants, the characteristics and the duration of the intervention, the outcome measures, and the results obtained.

### 2.4. Risk of bias

The authors independently carried out the selection of the papers, the extraction of the data, and their analysis. We used the Newcastle-Ottawa Scale (48) to assess the quality of the cohort and case-control studies and an adapted form of the Newcastle-Ottawa Scale for cross-sectional studies (49), checking characteristics such as randomization and blinding.

## 3. Results

### 3.1. Study selection

The articles included in this review were collected from PUBMED (24), SCOPUS (383), and Cochrane Library (44) databases. We enclosed 12 more studies from a manual review of the bibliography of the included articles. From these 465 articles, duplicates (6) and some papers after reading the title and abstract (395) were removed. Of 64 articles selected from the main text, 35 were rejected in accord with the exclusion criteria. Finally, we selected 29 articles for inclusion in this systematic review (Figure 1).

The studies included analyzed the association between cognitive impairment and consumption of alcohol (36, 50–77), coffee (36, 50, 52, 57, 58, 68, 73, 76, 77), cocoa (36, 52, 58) and soy-based foods (72, 73, 76, 77). Some of these articles studied the effects on cognition of fermented foods and beverages as part of a diet (36, 72–76) and will be discussed in a separate section.

### 3.2. Study characteristics

Of the 29 selected studies, 22 were cohort studies (52–56, 58–62, 64–66, 68–75), 4 case-control (36, 57, 63, 67) and 3 (51, 76, 77) cross-sectional studies. All the articles were published in English.

The possibility of doing a meta-analysis was rejected because of the high heterogeneity of the data. We found many differences in the included articles in clinical aspects (participants: number, sex, age; intervention: consumption of wine, beer, or alcohol in general, individual food or integrated in a diet, etc.); methodology (design of the studies and the risk of bias); and statistical processes (different statistical analysis).

To evaluate the quality of these articles, we used the Newcastle-Ottawa Scale (48, 49). All articles included in this review scored at least 7 points on the Newcastle-Ottawa Scale.

Information about the design, quality, and intervention of the included studies is shown in Table 1.

**Table 1:** Characteristics of the included publications.

**Author, year**	**Study name/cohort, country**	**Type of study**	**NOS score**	**Age (% of females)**	**Follow-up time**	**Adjustment for possible covariates**	**Intervention of interest (consumption of fermented products)**
Fischer et al. (50)	German study on aging, cognition and dementia in primary care patients, Germany	Cohort study	9	81.2 ± 3.4 (65.3%)	10 years	Age, sex, BMI, education, APOE4 carrier status, smoking status, physical activity score, depression, hypercholesterolemia, and a modified Charlson comorbidity disease index	Red wine, white wine, and coffee
Gu et al. (51)	The Washington heights-inwood Columbia aging project (WHICAP), USA	Cross-sectional study	10	80.1 ± 5.5 (67%)	1.5 years	Age, education, caloric intake, BMI, ethnicity, sex, APOE4 carrier status, smoking status, and history of diabetes, hypertension, heart disease, and clinical stroke.	Wine, beer, and liquor
Haller et al. (52)	Community-based population living in Geneva and Lausanne, Switzerland	Cohort study	8	73.8 ± 3.5 (55.9%)	3 years	Age, sex, education level, and Mini-Mental State Examination values.	Chocolate, wine, and coffee
Luchsinger et al. (53)	The Washington heights inwood-Columbia aging project (WHICAP), USA	Cohort study	9	73.3 ± 5.8 (67%)	4.1 ± 1.5 years	Age, sex, education, APOE4 carrier status, and heart disease	Wine and beer
Weyerer et al. (54)	Six centers of primary care, Germany	Cohort study	9	>75 (65.41%)	3 years	Age, sex, education level, instrumental activities of daily living impairment, living situation, somatic co-morbidity, APOE4 carrier status, smoking status, depression, and mild cognitive impairment	Alcohol, wine, and beer
Larrieu et al. (56)	PAQUID study, France	Cohort study	7	>65 (NE%)	8 years	Age, sex, and educational level	Wine
Lin et al. (77)	Nutrition and health survey in Taiwan (NAHSIT 2005–2008), Taiwan)	Cross-sectional study	10	73.3 ± 6.0 (49.4%)	3 years	Age, sex, educational level, soy-based foods intake, and physical component summary	Soybean, coffee, alcohol
Lindsay et al. (57)	Canadian study of health and aging (CSHA), Canada	Case-control study	8	≥70 (57.54%)	5 years	Age, sex, and education	Alcohol, wine, and coffee
Corley et al. (55)	Lothian birth cohort 1936, Scotland	Cohort study	7	69.5 ± 0.8 (51.7%)	2–3 months	Marital status, education level, smoking status, and medical history	Alcohol, wine, sherry-port, and beer
Low et al. (36)	3C study, France	Case-control study	9	76–78 (66%)	8.5 years (mean)	BMI, diabetes, fasting plasma levels of glucose, cholesterol, and triglycerides, APOE4 genotype, lifestyle factors, and smoking status	22 metabolites, including coffee, cocoa, alcohol, and wine
Paganini-Hill et al. (58)	The 90+ study (Leisure World Cohort Study), USA	Cohort study	8	93 ± 2.6 (^a^ %)	3 years	Age, sex, and education	Alcohol, coffee, and chocolate
Broe et al. (59)	Sydney older persons study, Australia	Cohort study	8	75–96 (49.5%)	3 years	Age, sex, and years of education	Alcohol
Huang et al. (60)	Kungsholmen project, Sweden	Cohort study	8	>75 (81%)	6 years	Age, sex, education, smoking, and institutionalization	Alcohol
Ogunniyi et al. (61)	African American cohort and Nigeria (Yoruba cohort), USA	Cohort study	8	African American cohort: 77.4 ± 6.4 (69.9%); Yoruba cohort: 75.6 ± 6.7 (63.5%)	5 years	Age, sex, years of education, and demographic, lifestyle, medical, and family history items	Alcohol
Järvenpää et al. (62)	Finnish Twin cohort study, Finland	Cohort study	8	74.6 ± 5.97 (55%)	25 years	Age, sex, and education level	Alcohol
Hébert et al. (63)	Canadian study of health, and aging working group, Canada	Case-control study	7	≥65 (58.10%)	5 years	Age and region	Alcohol, beer, and wine
Cervilla et al. (64)	Gospel Oak in London, UK	Cohort study	9	≥65 ^a^	1 year	Age, sex, occupational class, education, handicap status, depression and baseline cognitive function, and smoking and alcohol before and after age of 65	Alcohol
Stampfer et al. (65)	The nurses' health study, USA	Cohort study	8	70–81 (100%)	1.3–5.5 years	Age and education level	Alcohol
Espeland et al. (66)	The women's health initiative memory study (WHIMS), USA	Cohort study	7	50–79 (100%)	4, 2 years (mean)	Age, number of years since menopause, education, ethnicity, family income, smoking status. BMI, hypertension status, prior cardiovascular disease, diabetes, statin use, aspirin use, and prior hormone therapy.	Alcohol
Anttila et al. (67)	Cardiovascular risk factors, aging, and dementia (CAIDE) study, Finland	Case-control study	8	71.7 ± 4.1 (62%)	23 years (mean)	Age, sex, education, follow-up time, body mass index, total serum cholesterol, systolic blood pressure, diastolic blood pressure, smoking, history of myocardial infarction, and history of stroke	Alcohol
Vercambre et al. (68)	E3N cohort, France	Cohort study	7	76–82 (100%)	13 years	Age, education level, smoking status. BMI, physical activity, dietary energy intake, and medical history	33 macro- or micronutrients including dairy products, bread, coffee, and alcoholic drinks (beer and wine)
Ganguli et al. (69)	Monongahela Valley Independent Elders Survey (MoVIES project), USA	Cohort study	8	74.6 ± 5.34 (60.8%)	7 years	Age, sex, educational level, recruitment status, smoking, and depressive symptoms.	Alcohol
Launer et al. (70)	The Zutphen Elderly Study, Netherlands	Cohort study	7	65–84 (0%)	3 years	Age, education, and smoking status	Alcohol
Lemeshow et al. (71)	PAQUID Study, France	Cohort study	9	≥65 ^a^	3 years	Age	Wine
Lefèvre-Arbogast et al. (72)	3C study, France	Cohort study	9	75.8 ± 4.8 (62.1%)	12 years	Sex, education level, alcohol and tobacco consumption, regular physical activity, APOE4 carrier status, cardiovascular risk factors, comorbidities, and depressive symptoms	Red wine and soy products
Chen et al. (73)	National Taiwan University Hospital, Taiwan	Cohort study	7	≥65 ^a^	2 years	Age, sex, years of education, APOE4 carrier status, and supplement use (e.g., multivitamin and calcium)	Alcohol, bread, coffee, dairy (milk and cheese), fermented foods (miso and fermented bean curd), tea (semi-fermented and fermented tea)
Tangney et al. (74)	Chicago Health and Aging Project (CHAP), USA	Cohort study	8	75.4 ± 6.2 (61.7%)	3 years	Age, sex, race, education level, participation in cognitive activities, and total energy intake	Mediterranean-type diet (wine, alcohol, and breads); HEI-2005 (milk and milk-products, dark breads, beer, wine, and liquor)
Morris et al. (75)	Rush Memory and Aging Project (MAP), USA	Cohort study	8	81.4 ± 7.2 (75%)	4.7 years	Total energy intake, age, education, APOE4, smoking history, cognitive activities, physical activity, depressive symptoms, body mass index, hypertension, diabetes, heart disease history, and clinical stroke history	Mediterranean-DASH Diet Intervention for Neurodegenerative Delay (MIND diet, including dairy products and wine)
Okubo et al. (76)	SONIC study, Japan	Cross-sectional study	8	69–71 (54%)	1 month	Socioeconomic status, psychosocial variables, medical conditions of self and family, dental conditions, diet, and lifestyle; medical and physical examinations	58 food items including bread; milk and milk products, soy products, miso soup, alcoholic beverages (beer, sake, shochu, and wine), black and oolong tea, and coffee

AD, Alzheimer's disease; APOE4, E4 allele of the Apolipoprotein E gene; BMI, body mass index; DASH, Dietary Approach to Systolic Hypertension; HEI-2005, Healthy Eating Index-2005. ^*a*^NE = non specified.

To determine the cognitive performance, the outcome measures were examined neuropsychologically and clinically, including brain MRI scans (51, 52)—taking into account anatomical aspects such as total brain volume size, white matter preservation or cerebral blood flow-and ordinary validated cognitive scales for assessing different domains of cognition (general cognitive ability, episodic memory, semantic memory, working memory, verbal memory, etc.), such as a mini-mental state examination (MMSE), a structured interview for the diagnosis of dementia of the Alzheimer's disease type (SIDAM), the diagnostic and statistical manual of mental disorders (DSM) and variations, the short portable mental status questionnaire (SPMSQ), National Institute of Neurological and Communicative Disorders and Stroke (NINCDS), clinical dementia rating scale (CDR), and the Consortium to Establish a Registry for Alzheimer's Disease (CERAD) tests, etc. (36, 53–77).

### 3.3. Fermented products consumption and cognitive function

The effect of the consumption of fermented products either as individual items (36, 50–71, 77) or integrated into a diet (72–76) was analyzed in the articles included. To avoid possible confusion due to food interactions, the studies in the latter category have been excluded from the overall analysis and will be discussed in a separate section.

#### 3.3.1. Alcohol consumption

The consumption of alcohol–particularly wine and beer–was examined by most of the articles included in this review (50–71, 77). The main results obtained, and their statistical values are shown in Table 2.

**Table 2:** Cognitive effects observed following alcohol consumption.

**Author, year**	**Population exposed to intervention/total population (percentage)**	**Fermented beverage consumption**	**Quantity of ethanol intake (g/day)**	**Sex**	**Cognitive effects**
Fischer et al. (50)	19, 665/2,622 (7.5%)	Red win	≥14 g/day (≥1 drink/day)	Both	Lower incidence of AD [HR (95% CI) = 0.92 (0.85–0.99); *p =* 0.045]
	10,374/910 (11.4%)			Men	Lower incidence of AD [HR (95% CI) = 0.82 (0.74–0.92); *p < * 0.001]
	9,245/1,712 (5.4%)			Women	Higher incidence of AD [HR (95% CI) = 1.15 (1.00–1.32); *p =* 0.044]
	23,598/2,622 (1.9%)	White wine		Both	Not significantly associated with AD [HR (95% CI) = 1.00 (0.91–1.12); *p =* 0.875]
	2,366/910 (2.6%)			Men	Not significantly associated with a more rapid memory decline [B (95% CI) = 0.04 (−0.09–0.17); *p =* 0.562]
	2,568/1,712 (1.5%)			Women	More pronounced (not statistically significant) decline in memory over time [B (95% CI) = −0.13 (−0.26–0.001); *p =* 0.052]
	1,102/551 (2%)			Both- APOE4 carriers	Significantly associated with the incidence of AD in APOE4 carriers [HR (95% CI) = 1.21 (1.01–1.46); *p =* 0.044]
Gu et al. (51)	Light to moderate consumers: 180/589 (30.56%)	Alcohol (any type), wine, and beer	≤20 g/day in men (≤2 drinks/day) and ≤10 g/day in women (≤1 drink/day)	Both	Larger total brain volume [total alcohol: β = 0.007. *p =* 0.04; wine: β = −0.008. *p =* 0.05; beer: β = 0.002. *p =* 0.67]
Haller et al. (52)	Light consumption: 53/145 (36.6%); moderate consumption: 57/145 (39.3%); and heavy consumption: 35/145 (24.1%)	Wine	Light drinkers: ≤3, 7 g/day (0–8 drinks/month)	Both	Increased consumption of wine was related to an unfavorable cognitive evolution [OR adjusted (95% CI) = 1.012 (1.001–1.022); *p =* 0.028]. Light wine drinkers developed less white-matter lesions (*p < * 0.05) in all individuals and a better blood-flow in cognitively stable individuals (*p < * 0.05)
Luchsinger et al. (53)	138/980 (14.08%)	Wine	0.46-42 g/day (1–3 drinks/day)	Both	Lower risk of AD [HR (95% CI) = 0.55 (0.34–0.89); *p =* 0.015] and of dementia (not statistically significant) [HR (95% CI) = 0.42 (0.15–1.15); *p =* 0.091]
	139/980 (14.18%)	Beer			Lower risk (not statistically significant) of dementia but not of AD [Dementia: HR (95% CI) = 0.69 (0.26–1.83); *p =* 0.450; AD:HR (95% CI) = 1.47 (0.98–2.22); *p =* 0.065]
Weyerer et al. (54)	356/3,180; (11.19%)	Alcohol (any type)	20–29 g/day (1–2 drinks/day)	Both	Lower incidence of dementia [HR (95% CI) = 0.35 (0.17–0.69); *p =* 0.003] and AD [HR (95% CI) = 0.14 (0.03–0.56); *p =* 0.006]
	773/3,180 (24.30%)	Wine			Lower incidence (not statistically significant) of dementia [HR (95% CI) = 0.79 (0.55–1.13); *p =* 0.196] and AD [HR (95% CI) = 0.76 (0.46–1.23); *p =* 0.259]
	461/3,180 (14.49%)	Beer			Lower incidence (not statistically significant) of dementia [HR (95% CI) = 0.87 (0.56–1.35); *p =* 0.528] and AD [HR (95% CI) = 0.60 (0.30–1.21); *p =* 0.152]
Larrieu et al. (56)	Moderate consumers: 383/2,950 (13%)	Wine	42–56 g/day (3–4 drinks/day)	Both	Lower risk of dementia [RR (95% CI) = 0.56 (0.36–0.92)] and AD [RR (95% CI) = 0.53 (0.30–0.95)]
Lin et al. (77)	Moderate consumers: 254/1,105 (22.1%)	Alcohol^b^	Moderate^b^	Both	Negatively correlated with cognitive impaired [OR (95% CI) = 0.32 (0.17–0.61); *p < * 0.05]
Lindsay et al. (57)	Alcohol consumers (any type): 1,639/3, 985 (41.13%); Wine consumers: 683/3, 975 (17.18%)	Alcohol (any type) and wine	≥2 g/day (≥1 drink/week)	Both	Reduced risk of AD [alcohol: OR adjusted (95% CI) = 0.68 (0.47–1.00); wine: OR adjusted (95% CI) = 0.49 (0.28–0.88)]
Corley et al. (55)	Moderate consumers: 286/917 (31.2%)	Alcohol (any type), wine, and beer	Moderate level drinking:>28 g/day (>2 drinks/day)	Both	Better memory in women (0.46 ± 0.88; *p =* 0.002) and men (0.24 ± 0.93; *p < * 0.001) and performance (not statistically significant) on cognitive tests after alcohol intake (29 ± 1.2; *p =* 0.712)
	Moderate consumers: 208/442 (47.06%)			Men	Better verbal ability and memory with wine consumption (NART: *p < * 0.001; WTAR: *p =* 0.001; memory: *p =* 0.037) but not beer (NART: *p =* 0.07)
	Moderate consumers: 78/475 (16.42%)			Women	Positive association between wine intake and memory (*p =* 0.024) and verbal ability (NART: *p =* < 0.001 and WTAR: *p =* < 0.001)
Paganini-Hill et al. (58)	Consumers <2 drinks/day: 378/587 (44.29%); consumers >2 drinks/day: 120/587 (13.46%)^a^	Alcohol (any type)	<14 g/day (<2 drinks/day) - >14 g/day (>2 drinks/day)	Both	Beneficial effects with intake of <2 drinks/day [HR (95% CI) = 0.97 (0.73–1.28); *p <* 0.05] but not with >2 drinks/day [HR (95% CI) = 1.09 (0.75–1.58); *p <* 0.05]
Broe et al. (59)	-^b^	Alcohol (any type)	8, 43 g/day (<1 drink/day)	Both	Poorer visual reproduction I (*p <* 0.01)
Huang et al. (60)	Light-moderate consumers: 205/402 (50.61%)	Alcohol (any type)	Men: 1, 14–24 g/day (1–21 drinks/week); Women: 1, 14–16 g/day (1–14 drinks/week)	Both	Decreased risk of all dementia and AD [RR (95% CI) = 0.5 (0.3–0.7)]
Ogunniyi et al. (61)	Afro-Americans consumers: 147/470 (31.45%); Yoruba consumers: 120/523 (22.96%)	Alcohol (any type)	>20 g/day (>10 drinks/week)	Both	Protective effect in African Americans [OR (95% CI) = 0.49 (0.25–0.90); *p* ≤ 0.05]
Järvenpää et al. (62)	Binge drinking: 24/554 (4.33%)	Alcohol (any type)	Binge drinking (>5 bottles of beer/month or ≥1 bottle of wine/month on 1 occasion) or passing out (≥2 times/month)	Both	High risk of developing dementia [Binge drinking: OR (95% CI) = 4.2 (1.2–15); passing out [OR (95% CI) = 11.8 (3.3–42)] and cognitive decline [Binge drinking: OR (95% CI) = 2.4 (0.8–7.4)]; passing out: OR (95% CI) = 1.9 (0.3–11)]
Hébert et al. (63)	Beer consumers: 156/907 (17.19%); Wine consumers: 148/907 (16.32%)	Alcohol (any type), beer, and wine	≥2 g/day (1 drink/week)	Both	Protective effect for vascular dementia [Beer: OR (95% CI) = 0.66 (0.31–1.26); Wine: OR (95% CI) = 0.72 (0.34–1.39)]
Cervilla et al. (64)	Moderate consumers: 8/417 (1.92%)	Alcohol (any type)	Moderate drinking: 2–60 g/day (1–30 drinks/week)	Both	Non-significant trend of a protective effect for intake of 1–10 drinks/week [OR (95% CI) = 0.74 (0.2–2.1); *p =* 0.58] and 11–30 drinks/week [OR (95% CI) = 0.21 (0.1–1.9); *p =* 0.17]
Stampfer et al. (65)	Moderate consumers: 5,447/12,480 (43.65%)	Alcohol (any type)	Moderate: 1–14.9 g/day (1 drink/day)	Women	Better mean cognitive scores than for non-drinkers [Test of general cognition: RR (95% CI) = 0.77 (0.67–0.88); Global cognitive score: RR (95% CI) = 0.81 (0.70–0.93)] and less risk of cognitive decline after 2 years [RR (95% CI) = 0.85 (0.74–0.9.98)]
Espeland et al. (66)	≥1 drink/day consumers: 616/4,461 (13.8%)	Alcohol (any type)	>14 g/day (≥1 drink/day)	Women	Better scores in Modified Mini-Mental State Examination (*p <* 0.001) and in cognitive function [OR adjusted (95% CI) = 0.53 (0.28–10.99); *p =* 0.042]
Anttila et al. (67)	Never: 300/1,018 (25.47%); Infrequently: 423/1, 018 (41.55%); Frequently: 295/1,018 (28.98%)	Alcohol (any type)	Infrequently drinkers: <0, 47 g/day (<1 drink/month); frequently drinkers: >0.47 g/day (≥1 drink/month)	Both	Frequent drinkers [OR (95% CI) = 2.34 (1.15–4.77)] and never drinkers [OR (95% CI) = 2.08 (1.05–4.13)] had higher risk of mild cognitive impairment than infrequent drinkers; APOE4 presence enhanced the risk of dementia with increasing alcohol drinking [OR (95% CI) infrequent drinkers = 4.08 (0.98–16.91); OR (95% CI) frequent drinkers = 7.07 (1.37–36.60)]
Vercambre et al. (68)	-^b^	Wine	Mean: 9.03 g/day (4–5 drinks/week)	Women	Positive effect (not statistically significant) on recent cognitive decline [OR (95% CI) = 0.94 (0.75–1.18); *p =* 0.556] and on functional impairment [OR (95% CI) = 0.85 (0.68–1.04); *p =* 0.123]
		Beer	Mean: 0.46 g/day (<1 drink/week)		Trend of a positive effect on recent cognitive decline [OR (95% CI) = 0.86 (0.63–1.18); *p =* 0.459] but not on functional impairment [OR (95% CI) = 1.19 (0.91–1.56); *p =* 0.175]
Ganguli et al. (69)	Minimal drinking: 502/1,098 (45.72%); moderate drinking: 149/1,098 (13.57%)	Alcohol (any type)	Minimal drinking: <0.46 g/day (≤1 drink/month); moderate drinking: >0.47 g/day (>1 drink/month)	Both	Beneficial effects on cognitive decline (MMSE: [OR minimal drinkers: (95% CI) = 0.30 (0.14–0.65); [OR moderate (95% CI) = 0.08 (0.02–0.28); *p =* 0.05])
Launer et al. (70)	<1 drink/day consumers: 221/489 (45.19%); 1–2 drinks/day consumers: 122/489 (24.95%)	Alcohol (any type)	<13.2 g/day (<1 drink/day); 13.2 g/day-26.4 g/day (1–2 drinks/day)	Men	Significantly lower risk for poor cognitive function [ <1 drink/day: OR (95% CI) = 0.3 (0.2–0.7) and 1–2 drinks/day: OR (95% CI) = 0.2 (0.1–0.4)]
Lemeshow et al. (71)	≤1/4 liter/day consumers: 922/3,777 (24.41%); >1/4 liter/day consumers: 380/3,777 (10.06%)	Wine	Mild consumption: <24.69 g/day (<1/4 liter/day); Moderate-heavy consumption: >24.69 g/day (>1/4 liter/day)	Both	Protective effect with moderate-heavy consumption [OR unadjusted (95% CI) = 0.17 (0.06–0.48); OR adjusted (95% CI) = 0.23 (0.08–0.66)] but not with mild consumption [OR (95% CI) = 1.04 (0.61–1.78)]

AD, Alzheimer's disease; APOE4, E4 allele of the Apolipoprotein E gene; B, regression coefficient; β, regression beta coefficient; CI, confidence intervals; HR, Hazard Ratio; MRI, magnetic resonance imaging; NART, National Adult Reading Test; OR, Odds Ratio; SEE, standard error of estimate; WTAR, Wechsler Test of Adult Reading. ^*a*^Possible missing values; ^*b*^Non specified.

To facilitate the comparison between the different results, we have converted the alcohol intake quantities to grams of ethanol. In those articles where the ethanol intake was not specified in g, the conversion was made so that 1 drink (5 ounces of wine, 12 ounces of beer, or 1, 5 ounces of liquors) is equivalent to 14 g of alcohol (74).

The reviewed articles have found some beneficial cognitive effect after low-moderate alcohol consumption (1 drink/month-4 drinks/day) (50–58, 60, 61, 63–71, 77). Only in one case was no cognitive benefit observed, but a worse visual reproduction was reported following the ingestion of 8.43 g of alcohol/day (<1 drink/day) (59). This beneficial effect disappeared in carriers of the APOE4 allele (50, 53, 67) although an increase in the size of the total brain volume was observed in them (51). Heavy alcohol or binge consumption was associated with an increased risk of cognitive impairment, especially in women (50, 62, 68).

When stratifying by gender, some controversies were revealed. While some found a protective effect of alcohol in general (65, 66, 68), and for wine (55, 68) and beer (68) in women, others associated wine consumption in women with an increased risk of Alzheimer's disease or memory decline (50) or, in the case of beer, with functional impairment (68), so further studies are needed.

Specific analysis of the effects of wine consumption has shown that moderate consumption (from 1 drink per day up to 4) may reduce the risk of dementia and/or Alzheimer's disease and enhance cognitive functions (50, 53, 54, 56, 63, 68, 71) improve memory capabilities in women and verbal ability in men and women (55), and result in a larger total brain volume (≤2 drinks/day in men and ≤1 drink/day in women) (51). However, one study showed an unfavorable cognitive outcome, but better white-matter preservation and improved cerebral blood flow (52).

In relation to white wine, a higher intake (≥1 drink/day) was significantly associated with a higher incidence of Alzheimer's disease in APOE4 allele carriers (50).

Although some results are not statistically significant, moderate beer consumption (1–3 drinks/day) was related with a larger brain volume (51) and a trend toward a neuroprotective effect for dementia (53, 54, 63, 68), but it was also associated with poorer verbal ability in men (55). Regarding protection against Alzheimer's disease, some consider beer to have protective effects (54) and others do not (53).

Many of the articles reviewed also found cognitive benefits following consumption of low-moderate amounts of liquors (51, 55, 63, 68), such as memory performance in men (55), improvement in cognitive impairment (63, 68) and a tendency to increased total brain volume (51). These data are not shown in Table 2 because they do not specify the type of liquor, so we cannot include them as fermented beverages.

#### 3.3.2. Intake of coffee and cocoa

The effects of the intake of coffee (50, 52, 57, 58, 68, 77) and chocolate (52, 58) were also examined (Table 3). Tea consumption was excluded when the study was with non-fermented tea, the type used was not specified, or the tea consumption was integrated into a diet (73, 76).

**Table 3:** Cognitive effects observed following caffeine consumption.

**Author, year**	**Dietary intake of caffeine**	**Population exposed to intervention/total population (percentage)**	**Quantity ingested/day**	**Cognitive effects**
Fischer et al. (50)	Coffee	1,877/2,622 (71.6%)	≥115 mg of caffeine/day (≥1 cup of coffee)	Inverse associations (not statistically significant) were observed between higher intake and AD [HR (95% CI) = 0.97 (0.90–1.04); *p =* 0.338] and memory decline [B (95% CI) = −0.02 (−0.08–0.05); *p =* 0.241], also in APOE4 carriers [B (95% CI) = 0.11 (−0.06–0.29); *p =* 0.202] and APOE4 non-carriers [B (95% CI) = −0.04 (−0.11–0.02); *p =* 0.211]
Haller et al. (52)	Coffee	Moderate consumption: 47/145 (32.4%); Heavy consumption: 48/145 (33.1%)	Moderate consumption: 115–230 mg of caffeine/day (1–2 cups of coffee); Heavy consumption: 234–644 mg of caffeine/day (2–6 cups of coffee)	Moderate consumption: better cognitive performance [OR adjusted (95% CI) = 0.447 (0.210–0.952); *p =* 0.037]; moderate to heavy consumption: better bilateral deep white-matter preservation (*p <* 0, 05) and cerebral blood-flow in cognitively stable elderly (*p* < 0.05) in MRI
	Chocolate	Light consumption: 53/145 (36.6%); Moderate consumption: 46/145 (31.7%); Heavy consumption: 46/145 (31.7%)	Light consumption: 0–4 mg of caffeine/day (0–0.6 serving of chocolate); Moderate consumption: 4–16 mg of caffeine/day (0.7–2.6 servings); Heavy consumption: 16.2–45.2 mg of caffeine/day (2.7–7.5 servings)	Not associated with either cognitive outcomes or MRI parameters
Lin et al. (77)	Coffee	≥1 cup of coffee/day: 41/1, 105 (4.1%)	≥115 mg of caffeine/day (≥1 cup of coffee)	Beneficial effects on cognition for drinkers of ≥1 cup of coffee/day [OR unadjusted (95% CI) = 0.20 (0.04–0.98); *p <* 0.05]
Vercambre et al. (68)	Coffee	≥average consumption (1–2 cups of coffee)	≥230 mg of caffeine/day (≥2 cups of coffee)	Tendency of beneficial effects on cognitive decline [OR (95% CI) = 0.95 (0.71–1.28); *p =* 0, 804] but not on functional impairment [OR (95% CI) = 1.12 (0.84–1.50); *p =* 0.837]
Lindsay et al. (57)	Coffee	2,985/4,019 (74.27%)	≥115 mg of caffeine/day (≥1 cup of coffee)	Beneficial effects on cognition [OR (95% CI) = 0.69 (0.50–0.96]
Paganini-Hill et al. (58)	Coffee/chocolate	<50 mg/day: 156/587 (26.57%^a^); 50–199 mg/day: 198/587 (33.73%^a^); >200 mg/day: 138/587 (23.51%^a^)	Consumers of 50–199 mg of caffeine/day; Consumers of >200 mg of caffeine/day	Beneficial cognitive effects in 50–199 mg/day caffeine users [HR (95% CI) = 0.76 (0, 52–1, 10); *p <* 0.05] and in >200 mg/day caffeine users [HR (95% CI) = 0.66 (0.43–0.99); *p <* 0.05]

AD, Alzheimer's disease; APOE4, E4 allele of the Apolipoprotein E gene; CI, confidence intervals; HR, Hazard Ratio; MRI, magnetic resonance imaging; OR, Odds Ratio; SEE, standard error of estimate. ^*a*^Possible missing values.

Where data allowed, and to facilitate comparison between the different results, we estimated caffeine content (milligrams/standard unit) as 115 mg for regular coffee and 6 mg for chocolate (58).

All the results analyzed agree that the daily consumption of at least one cup of coffee was correlated with better cognitive performance (50, 52, 57, 58, 68, 77). Better preservation of bilateral deep white matter (*p* < 0, 05) and cerebral blood flow in cognitively stable elderly subjects (*p* < 0, 05) was also observed on MRI, with increasing consumption up to 5–6 cups (52). However, the neuroprotective role of chocolate is less clear in the reviewed articles (52, 58).

#### 3.3.3. Consumption of other fermented foods and beverages

Daily consumption of soy-based foods was inversely associated with cognitive impairment [OR (95% CI) = 0.45 (0.25–0.81); *p* < 0.01] (77). The same beneficial results were obtained when soya products were integrated into a diet (72, 73, 76) (see Section 3.3.4).

On the other hand, higher intakes of dairy desserts and ice cream were associated with higher odds of cognitive impairment [OR (95% CI) = 1.33 (1,07-1,65); *p* = 0.01] (68).

#### 3.3.4. Fermented products integrated into a diet

When fermented foods and beverages were integrated into a Mediterranean-type (74) or into a MIND diet—Intervention for neurodegenerative delay integrated for Mediterranean and DASH (dietary approach to systolic hypertension) diet (75)—rates of cognitive impairment decreased [(74): β = 0.014, SEE = 0.0004, *p* = 0.0004; (75): β = 0.0092; *p* < 0.0001].

Other dietary patterns including fermented foods and beverages—such as soy products, alcoholic beverages, coffee, etc.—were significantly associated with a better cognitive performance [βadjusted = 0.41 (95% CI):0, 17-0, 65; *p* < 0.001 (76)] and against decline of logical memory-recall [β = 0.18 (95% CI):0, 02-0, 33; *p* = 0.03 (73)]. Logical memory-recall decline was also improved in APOE4-carriers [OR (95% CI) = 0.71 (0.41–1.24); *p* = 0.23] and non-carriers [β (95% CI) = 0.18 (0.02–0.33); *p* = 0.03] with a dietary pattern rich in fermented foods (73).

Research on optimal polyphenol intake to reduce the risk of dementia and Alzheimer's disease by 50% proposes a dietary pattern (including for APOE4 carriers) that includes various fermented products [dementia: HR (95% CI) = 0.57 (0.37–0.86); *p* = 0.016; AD: HR (95% CI) = 0.54 (0.32–0.93); *p* = 0.045] (72).

Moreover, studying the relationship of diet-related metabolites with cognitive impairment showed two of the three biomarkers of coffee intake were inversely associated (atractyligenin glucuronide [OR = 0.72] and cyclo (leucyl-prolyl) [OR = 0.68]), but not that of the caffeine biomarker [OR = 1.75]. Cyclo (prolyl-valyl)—a metabolite found in chocolate and other fermented foods, e.g., beer, bread, cheddar cheese, cocoa, coffee, wine, Greek yogurt (78)—and an unidentified ion highly correlated with red wine and alcohol intake [OR = 0.69] were also inversely associated with cognitive impairment (36).

## 4. Discussion

Diet is increasingly used as a method of preventing disease and delaying aging. The use of fermented foods dates to prehistoric times, but findings on the benefits of this diet on the gut microbiota are relatively recent. The relationship between gut microbiota and the immune and nervous systems has been widely studied, and the implications of fermented food for mental processes performance, such as in dementia or Alzheimer's disease, are starting to be analyzed. For this reason, and considering that these are foods of daily consumption, it seems necessary to compile and analyze all the existing information on the elderly.

### 4.1. Strengths and weaknesses of this review

The main limitation encountered in conducting this review was the difference in information provided by the articles. It was difficult to compare results obtained in different populations, by different covariates, and using different methodologies. An attempt was made to solve this problem by converting fermented beverage intakes (amount in the form of drinks, cups, ounces, shots, or frequency of intake) to a standard measure (mg/day or g/day). Furthermore, the covariates for the different elements are diverse and may become very significant; for example, in the case of tobacco, which is associated not only with altered gut microbiota but also with gut inflammation (14). The variables in the papers were adjusted to these models to solve this problem.

The definition used of an elderly subject was a person aged 65 years or older (79–81), but perhaps the age range should have been extended to those under this age, since, as we have seen, the preventive effect of fermented products would occur in the long term.

In relation to the risk of bias, this review followed the preferred reporting items for systematic reviews and meta-analyses (PRISMA) guidelines (46) and the authors independently performed article selection, data extraction, analysis and assessed the quality of the included articles using the Newcastle-Ottawa scale (33, 34). All selected papers achieved the minimum quality criterion–i.e., 7 points on the NOS scale.

This work comprises a comprehensive review of current knowledge on the neuroprotective use of fermented foods and beverages. An important strength is that the protocol used to carry it out was registered and approved by the International Prospective Register of Systematic Reviews (PROSPERO) as CRD42021250921 (47).

### 4.2. Importance of a healthy gut microbiota

**Figure 2:**
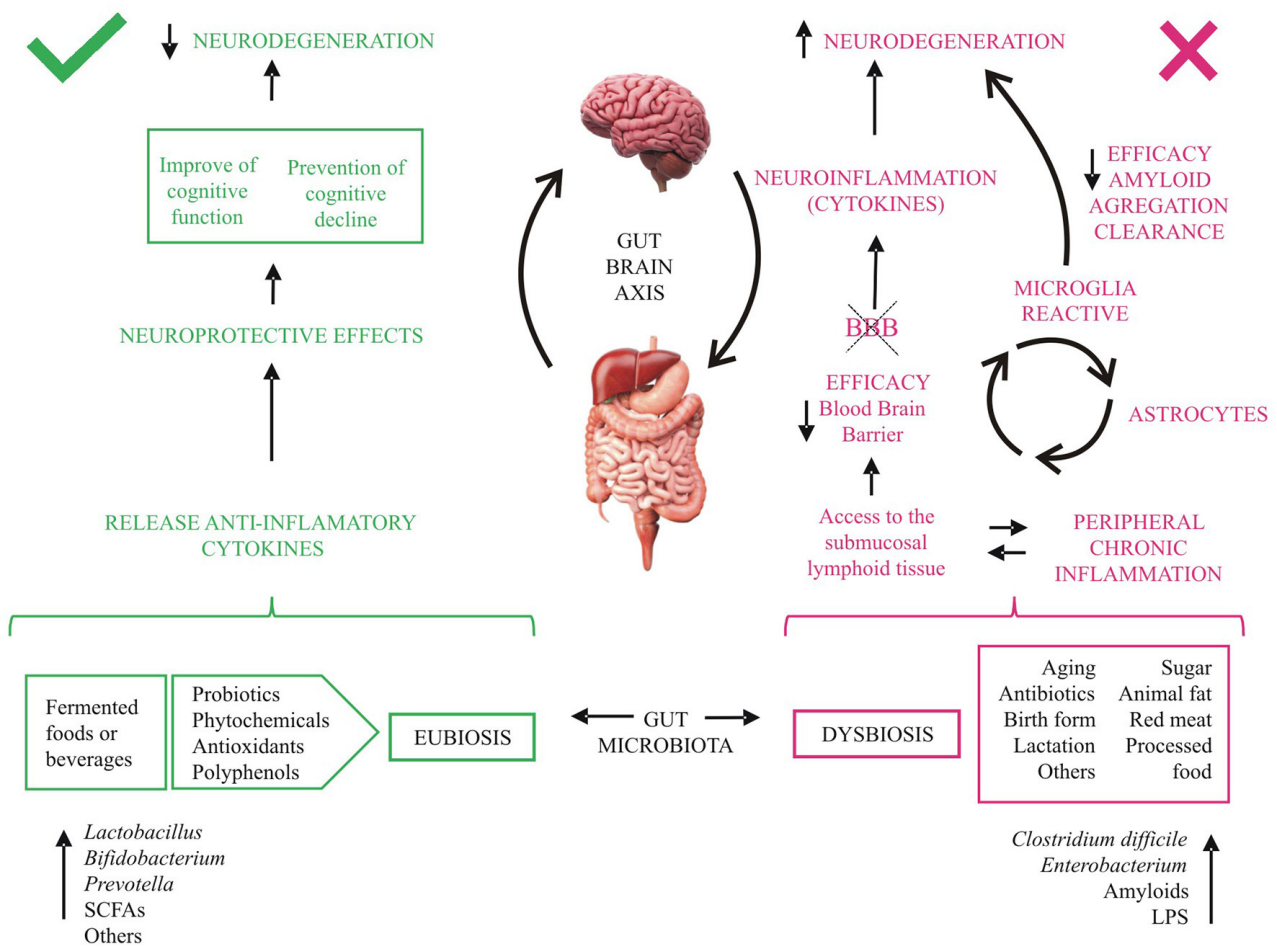
Effects of diet on cognitive decline. aa, amino acids; BBB, blood brain barrier; LPS, lipopolysaccharides; SCFAs, short- chain fatty acids. Fermented-food diet, ingestible microorganisms and others metabolites (green) act beneficially on intestinal health and inflammation and release anti-inflammatory cytokines, which promote neuroprotective effects. Several external factors, bacteria and components secreted by them (red) reduce over time the tightness of the intestinal barrier allowing access to the submucosal lymphoid tissue, favoring neuroinflammation and neurodegeneration.

The gut microbiota is essential to maintain the structural integrity of the intestinal mucosa and the immune system (see Section 1), but some diseases can disrupt the microbiota composition (1–3, 6, 12, 82). The balance in the gut microbiota can also be altered by diet, stress, antibiotic treatment, aging, and tobacco, among other factors (Figure 2). Pathogenic bacteria and their products such as lipopolysaccharides (LPS) may disrupt intestinal mucosal barriers, increasing the permeability of the blood-brain barrier. The increased permeability of the intestinal epithelial barrier results in an invasion of different bacteria, viruses, and their neuroactive products that support neuroinflammatory response in the brain (11). Alterations in the composition of the microbiota affect brain health due to the existence of the gut-brain axis, a complex bidirectional system involving the endocrine, immune, and nervous systems (3, 5, 6, 11, 12, 83).

#### 4.2.1. Implications of aging on gut microbiota

The changes in microbiome during aging involve a reduction in microbial biodiversity and metabolites, leading to a chronic inflammatory reaction and changes in the immune system (Figure 2). These processes generate a permanent activation of microglia that produces chronic inflammation and leads to damage and neuronal death, probably mediated by cytokines (2, 4–6, 12, 15, 28). Furthermore, during aging, the microglia activate astrocytes, worsening the neural inflammation and the blood-brain barrier dysfunction, reducing its efficiency in preventing the entrance of microorganism and other cells and metabolites into the central nervous system (4, 13–15). Moreover, gut microbes have been found to influence the maturation and function of microglia (11).

#### 4.2.2. The gut-brain axis in neurodegenerative diseases

A correlation has been found between an unhealthy gut microbiota and some nervous inflammatory and neurodegenerative diseases, such as some types of dementias and Alzheimer's disease (see Section 1).

Lately, the inflammatory hypothesis of Alzheimer's disease etiology is receiving attention (13, 14, 84–89). This involves the dysbiosis of the gut, mouth, and nose microbiota during aging, producing a systematic inflammatory response and the activation of the microglia to reduce it. Components secreted by bacteria reduce over time the impermeability of the intestinal barrier, enabling access to the submucosal lymphoid tissue. This phenomenon, together with deposits of amyloid-beta (Aβ) protein and hyperphosphorylated tau proteins, produces an inflammatory reaction, which impairs the blood-brain barrier, promoting neuroinflammation and neurodegeneration (13, 14, 43, 84). Accordingly, dysbiosis in the gut microbiota may provoke or enhance Alzheimer's disease, and it is possible that the structure of the gut microbiota is altered in Alzheimer's disease (13, 90).

Liu et al. identified the microbiome in patients with Alzheimer's disease and found its diversity was decreased. Moreover, this difference (and its importance) depended on the severity of the neurodegeneration. In individuals with Alzheimer's disease, the proportion of *Firmicutes* was significantly reduced, whereas that of *Proteobacteria, Gammaproteobacteria, Enterobacteriales*, and *Enterobacteriaceae* was increased, showing a progressively enriched prevalence from healthy individuals to mildly cognitive-impaired ones and patients with Alzheimer's disease (88).

The group of Harach generated germ-free mice using APPPS1 mice as an animal model of Alzheimer's disease (a double transgenic mice expressing mutated forms of the genes for human amyloid protein (APP) and presenilin 1) and found a drastic reduction of cerebral amyloid pathology when compared with control APP mice with intestinal microbiota. Furthermore, these amyloid deposits increased when germline mice were colonized with microbiota from APP transgenic mice more than when they were colonized with microbiota from wild-type mice (89).

In another mouse model of Alzheimer's disease, a dysregulated gut microbiota associated with the onset and progression of Alzheimer's disease was also observed, indicating that dysbiosis may occur before significant clinical signs appear, as evidenced by early alterations in short-chain fatty acids, compatible with intestinal inflammation (87).

The inefficiency of the microglia in reducing the amyloid deposits could explain the relationship between Alzheimer's disease and neuroinflammation (14, 15). Furthermore, pathogenic bacteria that are present in the gut microbiota due to dysbiosis can release amyloids, forming amyloid deposits in the brain. These amyloid deposits activate microglia, which fail to remove them, and participate in the production of proinflammatory cytokines, thus worsening neuroinflammation and initiating neurodegeneration (13, 15).

Choline supplementation has recently been found to restore a healthy cellular lipid status by promoting phospholipid synthesis in APOE4 cells *in vitro* and even in human APOE4 astrocytes (32). Moreover, women with APOE4 genotype have a higher risk of Alzheimer's disease (91, 92) and they tend to develop choline deficiency (93). The supplementation with citicoline improved cognitive performance, cerebral blood perfusion, and the brain bioelectrical activity pattern in Alzheimer's disease patients (94); fermented products contain choline, such as germs, soy, and milk (93).

In addition, several studies associate the dysregulation of serotonin and kynurenine route of tryptophan pathways, due to an alteration in the microbiota, with some neurodegenerative diseases, including Alzheimer's disease (15). Zhu proposes that tryptophan intake through the diet reduces central nervous system inflammation by decreasing astrocyte and microglial pathogenic activity (3, 95) and experiments in mice with multiple sclerosis showed dietary tryptophan to be neuroprotective (95). In this regard, tryptophan, and other derivatives in the kynurenine pathway (i.e., kynurenine, kynurenic acid, niacin, and nicotinamide) have been found in fermented food products: bread, beer, red wine, white cheese, yogurt, kefir, and cocoa (96).

### 4.3. Fermented diet as a possible preventive treatment in aging and in neurodegenerative diseases

Aging appears to be responsible for increasing the production of free radicals—enhancing brain oxidative stress—and for decreasing the activity of protective antioxidant enzymes. Thus, oxidative stress and vascular factors may also be involved in the development of dementia (56). In this regard, diets rich in antioxidant vitamins have shown cognitive improvements (97–99). Moreover, considering that nutrients—and their metabolites—can change the composition of the gut microbiota, a therapy focused on a diet recovering gut microbiota- such as the fermented diet- could have benefits in cognitive processes (4, 13, 15, 16, 100).

Fermented products are rich in probiotics and prebiotics. Probiotics are live microorganisms found naturally in some fermented foods. By consuming these foods, probiotics reach the gut, helping to balance the intestinal flora and improve digestion. Prebiotics are a type of food that is not fully digested by our bodies but can be fermented by beneficial bacteria in the gut, which contributes to their growth and development. This activity in the gut can affect the brain through two-way communication via the vagus nerve (90, 101).

The group of Hayden showed the diets with higher pro-inflammatory capacity were associated with a higher risk of mild cognitive impairment or dementia (102). Therefore, ingestible microorganisms present in fermented food act beneficially on intestinal health and inflammation, due to their positive effect on existing flora, as well as the release of anti-inflammatory cytokines (103, 104). Fermented-food diets have a very interesting nutritional content due to the biosynthesis of B vitamins, essential fatty acids and amino acids, proteins, and to the fact that they reduce anti-nutritional and toxic components and increase digestibility–as in the case of dairy products (reducing lactose content) and in legumes (reducing flatulence) (105–108).

Fermentation processes also enrich the bioactive peptides and create phytochemicals that can enhance the neuroprotective effects. Components modified through fermentation may improve bioavailability at the level of intestinal absorption and utilization of ingested nutrients and modulate the release of neurotransmitters such as brain-derived neurotrophic factor (BDNF), gamma-aminobutyric acid (GABA), and serotonin, which are involved in learning and memory processes (109). The study with FOS and GOS prebiotics by Savignac's group attributes its neuroprotective effects to increased BDNF levels in the dentate gyrus of the hippocampus in rats (42).

Moreover, fermented products such as tea, coffee, red wine, and cocoa products are rich in polyphenols—flavonoids, non-flavonoids (resveratrol), and phenolic acids. These bioactive compounds are being analyzed for their antioxidant properties and their possible role in intestinal permeability (2, 4, 16, 17, 36–38, 110) and their neuroprotective effects (2, 7, 8, 11, 26, 27, 111–114), even specifically in dementia and Alzheimer's disease (7, 8, 26, 27, 112, 113, 115). These effects could be due to their antioxidant, anti-inflammatory properties, and their ability to enhance the action of neurotrophic factors –among others, by increasing their concentration and/or the expression of tropomyosin receptor kinase (Trk) receptors (116, 117) or extracellular signal-regulated kinase and CREB pathways (117). Recent studies support the neuroprotective effect of resveratrol (13, 26), while others associate flavonoids with a lower risk of cognitive impairment (102, 118, 119). These authors argue that the mechanism by which flavonoids are neuroprotective appears to be the modification of the gut microbiota, specifically by increasing the proportion of *Bifidobacterium, Prevotella*, and *Lactobacillus* (17, 119, 120).

In any case, not all fermented foods contain polyphenols, such as dairy products. The concentration of polyphenols and their gastrointestinal absorption vary depending on the fermentation process; they are not useful if the intestinal microbiota is not healthy and is not able to break them down properly; and not all of them affect the colonic microflora and its fermentative capacity (121).

#### 4.3.1. Potential benefits of fermented alcohol beverages in delaying cognitive decline

Most of the reviewed articles found cognitive benefits in old adults following low-moderate alcohol consumption (36, 50–58, 60, 61, 63–71, 77). However, most of these benefits did not appear with compulsive intakes or in carriers of the APOE4 allele (50, 53, 62, 67, 68). Although some neuroprotective effects appear after moderate intake of beer (51, 53–55, 63, 68), the results obtained with wine (50–56, 63, 68, 71), especially in men and after red wine intake (50), are particularly promising.

Some authors claim vascular benefits after moderate alcohol consumption because it increases high-density lipoprotein (HDL) and decreases triglycerides and low- and very low-density lipoprotein (LDL, VLDL) levels, thus reducing the risk of vascular lesions (56, 61). That this occurs, not only with wine, but also with beer and liquors, could suggest a protective effect of alcohol itself on vascular factors (56).

This hypothesis is supported by the results of the articles included in this review that analyzed moderate consumption of liquor (not shown), which indicated a positive effect on memory performance in men (55) and on cognitive function (63, 68) and a tendency to increase total brain volume (51). Only in one case was it associated with a higher risk of dementia and Alzheimer's disease, though the results were not statistically significant (53).

On the other hand, the health benefits of alcohol consumption may be due not only to the fermentation process itself, but also to the food matrix, fermentation process and microorganisms, and to the content of polyphenols (resveratrol, anthocyanins, flavonoids, and catechins), vitamins, and other metabolites (see Section 4.3). That this occurs especially in the case of red wine could be explained by the fact that red wine is richer in resveratrol than white wine due to the different production processes, although the concentration varies according to climate, soil, and fermentation time of the wine (114, 122). This also happens with beer (for the flavonoid xanthohumol and its metabolites), but not with liquors, which—with some exceptions—have a lower concentration of polyphenols and lower antioxidant activity (114, 122).

Fischer found differences in the effect of wine consumption in women and men, probably due to gender variations in alcohol metabolism. Women are more susceptible to alcohol toxicity, perhaps due to their smaller volume of distribution for ethanol, decreased first-pass metabolism or more rapid absorption, and more rapid metabolism of ethanol (114, 123). Furthermore, there are also inter-individual differences, as each subject can metabolize the alcohol differently (114).

Fischer also reported a higher incidence of Alzheimer's disease in APOE4 carriers with white wine consumption (50). These findings contradict those obtained in a recent *in vitro* study, which showed that natural compounds, including resveratrol, could modify the structure of APOE4 forms and thus ameliorate the pathogenic effects associated with Alzheimer's disease (115). Fischer's results could therefore be due to the low concentration of resveratrol in white wine.

#### 4.3.2. Neuroprotective effects of coffee and chocolate

We analyzed the cognitive effects of caffeine intake in the form of coffee (36, 50, 52, 57, 58, 77) or chocolate (36, 52, 58).

The articles included in this review showed a neuroprotective effect of coffee in the elderly from the consumption of one cup per day (36, 52, 57, 58, 77), but not of chocolate (36, 52, 58). As some authors advocate a protective effect of caffeine (36, 124), it should be considered that the caffeine content of chocolate is much lower than that of coffee and that the concentration of cocoa in chocolate may differ. Moreover, these neuroprotective effects may not be due only to caffeine (125); coffee and cocoa are rich in phytochemicals (caffeine, chlorogenic acid, flavonoids, non-flavonoids and catechins) that confer on them antioxidant, prebiotic, anti-inflammatory, antihypertensive, hypoglycemic, vasculoprotective, neurostimulating, and neuroprotective properties (124, 126). Flavonoids and their metabolites can cross the blood-brain barrier and have been localized in brain areas related to learning and memory, such as the hippocampus, cerebral cortex, cerebellum, and striatum, enhancing or even being responsible for their possible cognitive effects (111, 125, 127–130). In animal models of Alzheimer's disease, flavonoids reduce amyloid-beta protein oligomerization and modulate the brain-derived neurotrophic factor (BDNF) signaling pathway (131, 132). They also interact at the cellular level with signaling cascades involving protein and lipid kinases that lead to inhibition of apoptosis induced by neurotoxic agents, such as oxygen radicals, and promote neuronal survival and synaptic plasticity, but also improve blood-flow and angiogenesis in the brain (133, 134). However, co-morbidities and personal genetics may influence the potential benefits and risks of coffee and cocoa (135).

#### 4.3.3. Other fermented products

The results analyzed showed that daily consumption of soy-based foods was inversely associated with cognitive impairment (77). These results were like those from other articles in younger populations not included in this review (136), in mice (137, 138) and those obtained when soya products were integrated into a diet (72, 73, 76).

Soy is rich in phytoestrogens and isoflavones (daidzein and genistein), which exert an anti-inflammatory and antioxidant effect and inhibit the effects of mitochondrial apoptosis (139–141), elevating existing neuronal function, and boost neuronal regeneration (142, 143). In addition, because of their structural similarity, isoflavones can bind to estrogen receptors β (ERβ), which are abundant in the central nervous system, thus affecting brain activity (139). Soy phytoestrogens can modify the gut microbiota, thereby influencing the gut-brain axis. Isoflavone supplementation has also been shown to improve cognitive function (139, 144). The content of isoflavones has been found to be different in different types of soy product, and their bioavailability is higher in fermented products (141).

On the other hand, sweet dairy products were associated with higher odds of cognitive impairment (68) in line with another study performed in 55–75-year-old adults (145). This could be due to their high fat and sugar content, but this later study associated less cognitive impairment with consumption of whole-fat milk and dairy products (145). These results are in contradiction with other findings that advocate the neuroprotective effects of milk and dairy consumption on the risk of dementia (136, 146, 147), so more studies are needed.

#### 4.3.4. Fermented-food diets

Dietary patterns including fermented foods and beverages—such as soy products, alcoholic beverages, coffee, etc.—showed some benefits regarding cognitive function (21, 36, 72–75), also in APOE4-carriers (72, 73).

The neuroprotective effects after consumption of fermented products are mainly due to a decrease in inflammatory processes (103, 104) and an increased release of brain-derived neurotrophic factors (42, 109), which enhance neuronal survival and differentiation (43). In animal models of Alzheimer's disease, probiotics modified the expression of GABA receptors in some brain regions related to learning and memory -such as the hippocampus, amygdala, and prefrontal cortex- and has been observed an improvement in hippocampal functions (43). As well as a decrease in pro-inflammatory cytokines levels -IL-1α, IL-1β, IL-2, IL-12, interferon-γ and TNF- α- leading to a decrease in accumulation of Aβ protein, neuroinflammation and neurodegeneration processes (90, 137, 148, 149). Probiotics also improved learning and memory processes (43, 149–151) regulated by long-term potentiation (LTP) possibly due to an increase in presynaptic neurotransmitter release (151) and excitatory postsynaptic potential in hippocampus (149). It should be considered that the beneficial effects of probiotics and fermented diets may depend on several factors, such as strain, dose, duration, age, host physiology, etc. (43).

These benefits could be due not only to the fermentation process itself, but also to a synergistic effect of the joint consumption of fermented products and to their concentrations of polyphenol compounds and vitamins (114). All these reasons encourage the possibility of a preventive treatment of cognitive decline through this type of diet, considering that a high consumption of some of these products (wine, coffee, etc.) may have undesirable effects, especially in vulnerable population.

#### 4.3.5. Current implications and outlook

Due to the aging of the population, dementias and cognitive impairment are becoming an increasingly important public health problem. This carries a significant economic and psychological cost for which there is no etiological treatment, so the most effective approach is an early detection and prevention.

Eurostat predicts the population of the European Union in 2040 will be 524 million, of which the estimate of patients with Alzheimer's disease will be 13.1 million. By 2080, this figure is calculated to increase to 13.7 million individuals with Alzheimer's disease out of a total population of 520 million persons (152).

Of the total global burden of diseases, 23% is attributed to disorders in people aged 60 years and over. Of this expenditure, neurological and mental disorders in the elderly accounted for 6.6% in 2010 (153). Primary prevention in adults below this age would dramatically improve morbidity, mortality, and expenditure data related to these chronic diseases.

We have already mentioned the importance of a healthy gut flora in inflammatory reactions and to the immune system (see Sections 1 and 4.2). The involvement of the microbiota in pathologies, including neurodegenerative diseases, is increasingly accepted. Therefore, one strategy to prevent cognitive decline could be to take care of the composition of this microbiota through diet.

In accordance, studies have shown that fermented foods and beverages produce significant improvements in gut permeability and the barrier function (2, 16, 36–38). In this sense, the Mediterranean-type diet has proved to be a balanced and healthy diet, compared with others (2, 4, 83), due to its monounsaturated and polyunsaturated fatty acids, fiber and low-glycemic carbohydrates, and polyphenols and other antioxidants. This could be because it includes a wide variety of fermented foods and beverages, such as coffee, wine, dairy products, etc., as opposed to the higher consumption of processed foods, sweets, and red meat in other diets. Studies associate this fermented diet and its beneficial effects with an increase in *Lactobacillus* and *Bifidobacterium*, and a decrease in *Clostridium* and *Enterobacterium*, among others, in the gut microbiota, providing anti-inflammatory effects and cardiovascular and brain protection (2–4, 25, 83) (see Figure 2).

In terms of digestibility, fermented foods are often easier to digest than non-fermented ones (154) because the microorganisms included provide certain enzymes, such as cellulases, which cannot be synthesized by humans (155). For example, microbial cellulases hydrolyze cellulose into sugars, which are easily digestible by humans, and pectinases soften the texture of food and release sugars for digestion. In addition, fermentation is a very productive and energy-efficient preservation process. It requires minimal cooking times and reduces the need for refrigeration or other forms of food preservation technology (155). Besides, during the fermentation process, toxins and anti-nutritional compounds commonly found in fruits and vegetables can be removed or detoxified by the action of microorganisms (155). Lactic acid bacteria produce lactate and acetate, which reduce the pH of food and inhibit other pathogenic organisms. It can also produce ethanol, hydrogen peroxide, and bacteriocins, which kill or suppress the growth of food-borne pathogenic bacteria and enhance food preservation and safety (106).

The use of microorganisms to meet the world's growing demand for food has enormous scope and potential. Fermentation is a very sustainable method as it can rescue waste that would otherwise be unusable as food by changing the consistency of the product and making it digestible. This increases the range of raw materials available as nutrients through the efficient utilization of natural foods and available feedstocks (155). Microorganisms in fermented foods are potential sources of useful components, such as organoleptic properties, textures, and colors that offer a wide range of foods to the consumer. Moreover, fermentation can improve the taste and appearance of foods. The strong flavors of fermented products can enhance the taste of a monotonous and boring diet (105–108).

Altough more studies in humans are necessary, this dietary pattern gives hope for the prevention of neurodegenerative diseases, above and beyond the public health and economic savings it would bring and the impact it could have on the food market.

## 5. Conclusions

In this systematic review, we have presented an array of published articles investigating the effects on cognitive status due to the consumption of fermented foods and beverages in the elderly. We can draw some conclusions from the articles studied:

1. Beneficial cognitive effects have been found following a low-moderate alcohol consumption, except in carriers of the APOE4 allele.

2. Heavy alcohol or binge consumption is associated with an increased risk of cognitive impairment, especially in women.

3. Moderate wine consumption appears to reduce the risk of dementia and/or Alzheimer's disease, to improve cognitive function, cerebral blood flow, and white-matter preservation, and to increase total brain volume.

4. Consumption of at least one cup of coffee per day is associated with better cognitive performance. Higher intakes (2-6 cups per day) are related to better preservation of bilateral deep white matter and cerebral blood flow.

5. An improvement in cognitive function following the daily intake of soy products has been observed.

6. Dietary patterns that include fermented foods and beverages have been shown to decrease the rates of cognitive decline, even in carriers of the APOE4 allele.

7. Due to the fermentation process itself and to the polyphenolic compounds and other antioxidants and vitamins in fermented products, the fermented-food diet could become an effective, safe, and inexpensive preventive or cognitive enhancement strategy.

## Data availability statement

The original contributions presented in the study are included in the article/supplementary material, further inquiries can be directed to the corresponding author.

## Author contributions

EP-G: literature search and data analysis and writing. IF-E: literature search and data analysis. JG-G and JF-G: data analysis and writing—review and editing. All authors contributed to the study conception and design.

## References

[1] Bäckhed F Ley RE Sonnenburg JL Peterson DA Gordon JI. Host-bacterial mutualism in the human intestine. Science. (2005) 307:1915–20. 10.1126/science.1104816 15790844

[2] Singh RK Chang HW Yan D Lee KM Ucmak D Wong K . Influence of diet on the gut microbiome and implications for human health. J Transl Med. (2017) 15:1–17. 10.1186/s12967-017-1175-y 28388917 PMC5385025

[3] Zhu S Jiang Y Xu K Cui M Ye W Zhao G . The progress of gut microbiome research related to brain disorders. J Neuroinflammation. (2020) 17:1–20. 10.1186/s12974-020-1705-z 31952509 PMC6969442

[4] Bernardi S del Bo' C Marino M Gargari G Cherubini A Andrés-Lacueva C . Polyphenols and intestinal permeability: rationale and future perspectives. J Agric Food Chem. (2020) 68:1816–29. 10.1021/acs.jafc.9b02283 31265272

[5] Fung TC Olson CA Hsiao EY. Interactions between the microbiota, immune and nervous systems in health and disease. Interactions. (2017) 20:145–55. 10.1038/nn.4476 28092661 PMC6960010

[6] Silva YP Bernardi A Frozza RL. The role of short-chain fatty acids from gut microbiota in gut-brain communication. Front Endocrinol. (2020) 11:1–14. 10.3389/fendo.2020.00025 32082260 PMC7005631

[7] Wang D Ho L Faith J Ono K Janle EM Lachcik PJ . Role of intestinal microbiota in the generation of polyphenol-derived phenolic acid mediated attenuation of Alzheimer's disease β-amyloid oligomerization. Mol Nutr Food Res. (2015) 59:1025–40. 10.1002/mnfr.201400544 25689033 PMC4498582

[8] Leblhuber F Steiner K Schuetz B Fuchs D Gostner JM. Probiotic supplementation in patients with Alzheimer's dementia—An explorative intervention study. Curr Alzheimer Res. (2018) 15:1106–13. 10.2174/1389200219666180813144834 30101706 PMC6340155

[9] Vogt NM Kerby RL Dill-McFarland KA Harding SJ Merluzzi AP Johnson SC . Gut microbiome alterations in Alzheimer's disease. Sci Rep. (2017) 7:1–11. 10.1038/s41598-017-13601-y 29051531 PMC5648830

[10] Power SE O'Toole PW Stanton C Ross RP Fitzgerald GF. Intestinal microbiota, diet and health. Br J Nutri. (2014) 111:387–402. 10.1017/S0007114513002560 23931069

[11] Shimizu Y. Gut microbiota in common elderly diseases affecting activities of daily living. World J Gastroenterol. (2018) 24:4750–8. 10.3748/wjg.v24.i42.4750 30479462 PMC6235798

[12] Mirzaei R Bouzari B Hosseini-Fard SR Mazaheri M Ahmadyousefi Y Abdi M . Role of microbiota-derived short-chain fatty acids in nervous system disorders. Biomed Pharmacotherapy. (2021) 139:111661. 10.1016/j.biopha.2021.111661 34243604

[13] Pistollato F Sumalla Cano S Elio I Masias Vergara M Giampieri F Battino M. Role of gut microbiota and nutrients in amyloid formation and pathogenesis of Alzheimer's disease. Nutr Rev. (2016) 74:624–34. 10.1093/nutrit/nuw023 27634977

[14] Quigley EMM. Microbiota-brain-gut axis and neurodegenerative diseases. Curr Neurol Neurosci Rep. (2017) 17:6. 10.1007/s11910-017-0802-6 29039142

[15] Sochocka M Donskow-Łysoniewska K Diniz BS Kurpas D Brzozowska E Leszek J. The gut microbiome alterations and inflammation-driven pathogenesis of Alzheimer's disease—A critical review. Mol Neurobiol. (2019) 56:1841–51. 10.1007/s12035-018-1188-4 29936690 PMC6394610

[16] Marco ML Heeney D Binda S Cifelli CJ Cotter PD Foligné B . Health benefits of fermented foods: microbiota and beyond. Curr Opin Biotechnol. (2017) 44:94–102. 10.1016/j.copbio.2016.11.010 27998788

[17] Zhang Y Cheng L Liu Y Zhan S Wu Z Luo S . Dietary flavonoids: a novel strategy for the amelioration of cognitive impairment through intestinal microbiota. J Sci Food Agric. (2023) 103:488–95. 10.1002/jsfa.12151 35892267

[18] Chandra S Sisodia SS Vassar RJ. The gut microbiome in Alzheimer's disease: what we know and what remains to be explored. Mol Neurodegener. (2023) 18:7. 10.1186/s13024-023-00595-7 36721148 PMC9889249

[19] Koblinsky ND Power KA Middleton L Ferland G Anderson ND. The role of the gut microbiome in diet and exercise effects on cognition: a review of the intervention literature. J Gerontol Series A. (2022) 3:166. 10.1093/gerona/glac166 35977540 PMC9951060

[20] Strasser B Ticinesi A. Intestinal microbiome in normal ageing, frailty and cognition decline. Curr Opin Clin Nutr Metab Care. (2023) 26:8–16. 10.1097/MCO.0000000000000878 36102345

[21] Dahiya D Nigam PS. Antibiotic-Therapy-Induced Gut Dysbiosis Affecting Gut Microbiota-Brain Axis and Cognition: Restoration by Intake of Probiotics and Synbiotics. Int J Mol Sci. (2023) 24:74. 10.3390/ijms24043074 36834485 PMC9959899

[22] Sharma R Gupta D Mehrotra R Mago P. Psychobiotics: the next-generation probiotics for the brain. Curr Microbiol. (2021) 78:449–63. 10.1007/s00284-020-02289-5 33394083

[23] Minerbi A Gonzalez E Brereton NJB Anjarkouchian A Dewar K Fitzcharles MA . Altered microbiome composition in individuals with fibromyalgia. Pain. (2019) 160:2589–602. 10.1097/j.pain.0000000000001640 31219947

[24] Clos-Garcia M Andrés-Marin N Fernández-Eulate G Abecia L Lavín JL van Liempd S . Gut microbiome and serum metabolome analyses identify molecular biomarkers and altered glutamate metabolism in fibromyalgia. EBioMedicine. (2019) 46:499–511. 10.1016/j.ebiom.2019.07.031 31327695 PMC6710987

[25] Tomova A Bukovsky I Rembert E Yonas W Alwarith J Barnard ND Kahleova H. The effects of vegetarian and vegan diets on gut microbiota. Front Nutr. (2019) 6:47. 10.3389/fnut.2019.00047 31058160 PMC6478664

[26] Köbe T Witte AV Schnelle A Tesky VA Pantel J Schuchardt J-P . Impact of resveratrol on glucose control, hippocampal structure and connectivity, and memory performance in patients with mild cognitive impairment. Front Neurosci. (2017) 11:1–11. 10.3389/fnins.2017.00105 28326010 PMC5339301

[27] Witte A V Kerti L Margulies DS Flöel A. Effects of resveratrol on memory performance, hippocampal functional connectivity, and glucose metabolism in healthy older adults. J Neurosci. (2014) 34:7862–70. 10.1523/JNEUROSCI.0385-14.2014 24899709 PMC6608268

[28] Rogers GB Keating DJ Young RL Wong ML Licinio J Wesselingh S. From gut dysbiosis to altered brain function and mental illness: Mechanisms and pathways. Mol Psychiatry. (2016) 21:738–48. 10.1038/mp.2016.50 27090305 PMC4879184

[29] Stelzmann RA Norman Schnitzlein H Reed Murtagh F. An english translation of alzheimer's 1907 paper,??ber eine eigenartige erkankung der hirnrinde? Clinical Anatomy. (1995) 8:429–31. 10.1002/ca.980080612 8713166

[30] Innerarity TL Mahley RW. Enhanced binding by cultured human fibroblasts of apo-E-containing lipoproteins as compared with low density lipoproteins. Biochemistry. (1978) 17:1440–7. 10.1021/bi00601a013 206278

[31] Mahley R Bersot T LeQuire V Levy R Windmueller H Brown W. Identity of very low density lipoprotein apoproteins of plasma and liver Golgi apparatus. Science. (1970) 68:380–2. 10.1126/science.168.3929.380 4985196

[32] Sienski G Narayan P Bonner JM Kory N Boland S Arczewska AA . APOE4 disrupts intracellular lipid homeostasis in human iPSC-derived glia. Sci Transl Med. (2021) 13:4564. 10.1126/scitranslmed.aaz4564 33658354 PMC8218593

[33] McIntosh AM Bennett C Dickson D Anestis SF Watts DP Webster TH . The Apolipoprotein E. (APOE) gene appears functionally monomorphic in Chimpanzees (Pan troglodytes) *PLoS One*. (2012) 7:e47760. 10.1371/journal.pone.0047760 23112842 PMC3480407

[34] Strittmatter WJ Weisgraber KH Huang DY Dong LM Salvesen GS Pericak-Vance M . Binding of human apolipoprotein E to synthetic amyloid beta peptide: isoform-specific effects and implications for late-onset Alzheimer's disease. Proc Nat Acad Sci. (1993) 90:8098–102. 10.1073/pnas.90.17.8098 8367470 PMC47295

[35] Baldi S Mundula T Nannini G Amedei A. Microbiota shaping — the effects of probiotics, prebiotics, and fecal microbiota transplant on cognitive functions: a systematic review. World J Gastroenterol. (2021) 27:6715–32. 10.3748/wjg.v27.i39.6715 34754163 PMC8554405

[36] Low DY Lefèvre-Arbogast S González-Domínguez R Urpi-Sarda M Micheau P Petera M . Diet-related metabolites associated with cognitive decline revealed by untargeted metabolomics in a prospective cohort. Mol Nutr Food Res. (2019) 63:1–10. 10.1002/mnfr.201900177 31218777 PMC6790579

[37] Bell V Ferrão J Pimentel L Pintado M Fernandes T. One health, fermented foods, and gut microbiota. Foods. (2018) 7:195. 10.3390/foods7120195 30513869 PMC6306734

[38] Dimidi E Cox S Rossi M Whelan K. Fermented foods : definitions and characteristics, gastrointestinal health and disease. Nutrients. (2019) 11:1–26. 10.3390/nu11081806 31387262 PMC6723656

[39] Aslam H Green J Jacka FN Collier F Berk M Pasco J . Fermented foods, the gut and mental health: a mechanistic overview with implications for depression and anxiety. Nutr Neurosci. (2020) 23:659–71. 10.1080/1028415X.2018.1544332 30415609

[40] Reid G Younes JA Van der Mei HC Gloor GB Knight R Busscher HJ. Microbiota restoration: natural and supplemented recovery of human microbial communities. Nat Rev Microbiol. (2011) 9:27–38. 10.1038/nrmicro2473 21113182

[41] Perez-Pardo P Kliest T Dodiya HB Broersen LM Garssen J Keshavarzian A . The gut-brain axis in Parkinson's disease: possibilities for food-based therapies. Eur J Pharmacol. (2017) 817:86–95. 10.1016/j.ejphar.2017.05.042 28549787

[42] Savignac HM Corona G Mills H Chen L Spencer JPE Tzortzis G . Prebiotic feeding elevates central brain derived neurotrophic factor, N-methyl-d-aspartate receptor subunits and d-serine. Neurochem Int. (2013) 63:756–64. 10.1016/j.neuint.2013.10.006 24140431 PMC3858812

[43] Thangaleela S Sivamaruthi BS Kesika P Chaiyasut C. Role of probiotics and diet in the management of neurological diseases and mood states: a review. Microorganisms. (2022) 10:2268. 10.3390/microorganisms10112268 36422338 PMC9696277

[44] Varankovich NV Nickerson MT Korber DR. Probiotic-based strategies for therapeutic and prophylactic use against multiple gastrointestinal diseases. Front Microbiol. (2015) 6:685. 10.3389/fmicb.2015.00685 26236287 PMC4500982

[45] Binns N. Probiotics, Prebiotics and the Gut Microbiota. ILSI Europe (2013).

[46] Moher D Shamseer L Clarke M Ghersi D Liberati A Petticrew M . Preferred reporting items for systematic review and meta-analysis protocols. (PRISMA-P) 2015 statement. Syst Rev. (2015) 4:1. 10.1186/2046-4053-4-1 25554246 PMC4320440

[47] PROSPERO. (International prospective register of systematic reviews)- CRD42021250921. Available online at: https://www.crd.york.ac.uk/prospero/display_record.php?RecordID=250921 (accessed June 5, 2023).

[48] Wells GA, Shea, B, O'Connell, D, Peterson, J, Welch, V, Losos, M, Tugwell, P,. The Newcastle-Ottawa Scale. (NOS) for Assessing the Quality of Nonrandomised Studies in Meta-Analyses. (2015). Available online at: http://www.ohri.ca/programs/clinical_epidemiology/oxford.asp (accessed June 5, 2023).

[49] Herzog R Álvarez-Pasquin MJ Díaz C. del Barrio JL, Estrada JM, Gil Á. Are healthcare workers' intentions to vaccinate related to their knowledge, beliefs and attitudes? A systematic review. BMC Public Health. (2013) 13:154. 10.1186/1471-2458-13-154 23421987 PMC3602084

[50] Fischer K Melo van Lent D Wolfsgruber S Weinhold L Kleineidam L Bickel H . Prospective associations between single foods, alzheimer's dementia and memory decline in the elderly. Nutrients. (2018) 10:852. 10.3390/nu10070852 29966314 PMC6073331

[51] Gu Y Scarmeas N Short EE Luchsinger JA DeCarli C Stern Y . Alcohol intake and brain structure in a multiethnic elderly cohort. Clin Nutr. (2014) 33:662–7. 10.1016/j.clnu.2013.08.004 24011900 PMC4048329

[52] Haller S Montandon ML Rodriguez C Herrmann FR Giannakopoulos P. Impact of coffee, wine, and chocolate consumption on cognitive outcome and MRI parameters in old age. Nutrients. (2018) 10:1–13. 10.3390/nu10101391 30275380 PMC6212945

[53] Luchsinger JA Tang M-X Siddiqui M Shea S Mayeux R. Alcohol intake and risk of dementia. J Am Geriatr Soc. (2004) 52:540–6. 10.1111/j.1532-5415.2004.52159.x 15066068

[54] Weyerer S Schäufele M Wiese B Maier W Tebarth F van den bussche H . Current alcohol consumption and its relationship to incident dementia: Results from a 3-year follow-up study among primary care attenders aged 75 years and older age. Ageing. (2011) 40:456–63. 10.1093/ageing/afr007 21367764

[55] Corley J Jia X Brett CE Gow AJ Starr JM Kyle JAM . Alcohol intake and cognitive abilities in old age: the Lothian Birth Cohort 1936 study. Neuropsychology. (2011) 25:166–75. 10.1037/a0021571 21381824

[56] Larrieu S Letenneur L Helmer C Dartigues JF Barberger-Gateau P. Nutritional factors and risk of incident dementia in the PAQUID longitudinal cohort. J Nutr Health Aging. (2004) 8:150–4. Available online at: http://www.ncbi.nlm.nih.gov/pubmed/15129300 (accessed July 19, 2022). 15129300

[57] Lindsay J Laurin D Verreault R Hébert R Helliwell B Hill GB . Risk factors for Alzheimer's disease: a prospective analysis from the Canadian study of health and aging. Am J Epidemiol. (2002) 156:445–53. 10.1093/aje/kwf074 12196314

[58] Paganini-Hill A Kawas CH Corrada MM. Lifestyle factors and dementia in the oldest-old: the 90 + study. Alzheimer Dis Assoc Disord. (2016) 30:21–6. 10.1097/WAD.0000000000000087 25710250 PMC4561216

[59] Broe GA Creasey H Jorm AF Bennett HP Casey B Waite LM . Health habits and risk of cognitive impairment and dementia in old age: a prospective study on the effects of exercise, smoking and alcohol consumption. Aust N Z J Public Health. (1998) 22:621–3. 10.1111/j.1467-842X.1998.tb01449.x 9744220

[60] Huang W Qiu C Winblad B Fratiglioni L. Alcohol consumption and incidence of dementia in a community sample aged 75 years and older. J Clin Epidemiol. (2002) 55:959–64. 10.1016/S0895-4356(02)00462-6 12464371

[61] Ogunniyi A Hall KS Gureje O Baiyewu O Gao S Unverzagt FW . Risk factors for incident Alzheimer's disease in African Americans and Yoruba. Metab Brain Dis. (2006) 21:235–40. 10.1007/s11011-006-9017-2 16850256 PMC3199593

[62] Järvenpää T Rinne JO Koskenvuo M Räihä I Kaprio J. Binge drinking in midlife and dementia risk. Epidemiology. (2005) 16:766–71. 10.1097/01.ede.0000181307.30826.6c 16222166

[63] Hébert R Lindsay J Verreault R Rockwood K Hill G Dubois MF. Vascular dementia. Stroke. (2000) 31:1487–93. 10.1161/01.STR.31.7.1487 10884442

[64] Cervilla JA. Smoking, drinking, and incident cognitive impairment: a cohort community based study included in the Gospel Oak project. J Neurol Neurosurg Psychiatry. (2000) 68:622–6. 10.1136/jnnp.68.5.622 10766894 PMC1736927

[65] Stampfer MJ Kang JH Chen J Cherry R Grodstein F. Effects of moderate alcohol consumption on cognitive function in women. New England Journal of Medicine. (2005) 352:245–53. 10.1056/NEJMoa041152 15659724

[66] Espeland MA Gu L Masaki KH Langer RD Coker LH Stefanick ML . Association between reported alcohol intake and cognition: results from the women's health initiative memory study. Am J Epidemiol. (2005) 161:228–38. 10.1093/aje/kwi043 15671255

[67] Anttila T Helkala E-L Viitanen M Kåreholt I Fratiglioni L Winblad B . Alcohol drinking in middle age and subsequent risk of mild cognitive impairment and dementia in old age: a prospective population based study. BMJ. (2004) 329:539. 10.1136/bmj.38181.418958.BE 15304383 PMC516103

[68] Vercambre MN Boutron-Ruault MC Ritchie K Clavel-Chapelon F Berr C. Long-term association of food and nutrient intakes with cognitive and functional decline: a 13-year follow-up study of elderly French women. Br J Nutri. (2009) 102:419–27. 10.1017/S0007114508201959 19203415 PMC2891709

[69] Ganguli M Vander-Bilt J Saxton JA Shen C Dodge HH. Alcohol consumption and cognitive function in late life: a longitudinal community study. Neurology. (2005) 65:1210–7. 10.1212/01.wnl.0000180520.35181.24 16247047

[70] Launer LJ Feskens EJM Kalmijn S Kromhout D. Smoking, drinking, and thinking: the Zutphen elderly study. Am J Epidemiol. (1996) 143:219–27. 10.1093/oxfordjournals.aje.a008732 8561155

[71] Lemeshow S Letenneur L Dartigues JF Lafont S Orgogozo JM Commenges D. Illustration of analysis taking into account complex survey considerations: the association between wine consumption and dementia in the PAQUID study. Personnes Ages Quid Am J Epidemiol. (1998) 148:298–306. 10.1093/oxfordjournals.aje.a009639 9690368

[72] Lefèvre-Arbogast S Gaudout D Bensalem J Letenneur L Dartigues JF Hejblum BP . Pattern of polyphenol intake and the long-term risk of dementia in older persons. Neurology. (2018) 90:e1979–88. 10.1212/WNL.0000000000005607 29703769

[73] Chen YC Jung CC Chen JH Chiou JM Chen TF Chen YF . Association of dietary patterns with global and domain-specific cognitive decline in Chinese elderly. J Am Geriatr Soc. (2017) 65:1159–67. 10.1111/jgs.14741 28092399

[74] Tangney CC Kwasny MJ Li H Wilson RS Evans DA Morris MC. Adherence to a Mediterranean-type dietary pattern and cognitive decline in a community population. Am J Clin Nutr. (2011) 93:601–7. 10.3945/ajcn.110.007369 21177796 PMC3041601

[75] Morris MC Tangney CC Wang Y Sacks FM Barnes LL Bennett DA . diet slows cognitive decline with aging. Alzheimer's and Dementia. (2015) 11:1015–22. 10.1016/j.jalz.2015.04.011 26086182 PMC4581900

[76] Okubo H Inagaki H Gondo Y Kamide K Ikebe K Masui Y . Association between dietary patterns and cognitive function among 70-year-old Japanese elderly: a cross-sectional analysis of the SONIC study. Nutr J. (2017) 16:2. 10.1186/s12937-017-0273-2 28893250 PMC5594454

[77] Lin H-C Peng C-H Huang C-N Chiou J-Y. Soy-based foods are negatively associated with cognitive decline in Taiwan's elderly. J Nutr Sci Vitaminol. (2018) 64:335–9. 10.3177/jnsv.64.335 30381623

[78] Borthwick AD da Costa NC. 2,5-diketopiperazines in food and beverages: taste and bioactivity. Crit Rev Food Sci Nutr. (2017) 57:718–42. 10.1080/10408398.2014.911142 25629623

[79] Lohr KN. Medicare: a strategy for quality assurance. J Healthcare Qual. (1991) 13:10–3. 10.1111/j.1945-1474.1991.tb00115.x 10109548

[80] OECD. Elderly Population (indicator). OECD. (2021). 10.1787/8d805ea1-en

[81] Aged-MeSH,. NCBI. (1996). Available online at: https://www.ncbi.nlm.nih.gov/mesh/68000368 (accessed January 17, 2022).

[82] MacDonald TT Monteleone G. Immunity, inflammation, and allergy in the gut. Science. (2005) 307:1920–5. 10.1126/science.1106442 15790845

[83] Rinninella C Raoul L Scaldaferri P Miggiano G Mele. Food components and dietary habits: keys for a healthy gut microbiota composition. Nutrients. (2019) 11:2393. 10.3390/nu11102393 31591348 PMC6835969

[84] Wang WY Tan MS Yu JT Tan L. Role of pro-inflammatory cytokines released from microglia in Alzheimer's disease. Ann Transl Med. (2015) 3:136. 10.3978/j.issn.2305-5839.2015.03.49 26207229 PMC4486922

[85] Cameron B Landreth GE. Inflammation, microglia, and Alzheimer's disease. Neurobiol Dis. (2010) 37:503–9. 10.1016/j.nbd.2009.10.006 19833208 PMC2823849

[86] Agostinho PA Cunha R Oliveira C. Neuroinflammation, oxidative stress and the pathogenesis of Alzheimer's disease. Curr Pharm Des. (2010) 16:2766–78. 10.2174/138161210793176572 20698820

[87] Favero F Barberis E Gagliardi M Espinoza S Contu L Gustincich S . A Metabologenomic approach reveals alterations in the gut microbiota of a mouse model of Alzheimer's disease. PLoS ONE. (2022) 17:e0273036. 10.1371/journal.pone.0273036 36001607 PMC9401139

[88] Liu P Wu L Peng G Han Y Tang R Ge J . Altered microbiomes distinguish Alzheimer's disease from amnestic mild cognitive impairment and health in a Chinese cohort. Brain Behav Immun. (2019) 80:633–43. 10.1016/j.bbi.2019.05.008 31063846

[89] Harach T Marungruang N Duthilleul N Cheatham V Mc Coy KD Frisoni G . Reduction of Abeta amyloid pathology in APPPS1 transgenic mice in the absence of gut microbiota. Sci Rep. (2017) 7:1–13. 10.1038/srep41802 28176819 PMC5297247

[90] Kowalski K Mulak A. Brain-Gut-Microbiota Axis in Alzheimer's Disease. J Neurogastroenterol Motil. (2019) 25:48–60. 10.5056/jnm18087 30646475 PMC6326209

[91] Neu SC Pa J Kukull W Beekly D Kuzma A Gangadharan P . Apolipoprotein E genotype and sex risk factors for Alzheimer disease. JAMA Neurol. (2017) 74:1178. 10.1001/jamaneurol.2017.2188 28846757 PMC5759346

[92] Damoiseaux JS Seeley WW Zhou J Shirer WR Coppola G Karydas A . Gender modulates the APOE ε4 effect in healthy older adults: convergent evidence from functional brain connectivity and spinal fluid tau levels. J Neurosci. (2012) 32:8254–62. 10.1523/JNEUROSCI.0305-12.2012 22699906 PMC3394933

[93] Zeisel SH Da Costa KA. Choline: an essential nutrient for public health. Nutr Rev. (2009) 67:615–23. 10.1111/j.1753-4887.2009.00246.x 19906248 PMC2782876

[94] Alvarez XA Mouzo R Pichel V Pérez P Laredo M Fernández-Novoa L . Double-blind placebo-controlled study with citicoline in APOE genotyped Alzheimer's disease patients. Effects on cognitive performance, brain bioelectrical activity and cerebral perfusion. Methods Find Exp Clin Pharmacol. (1999) 21:633–44. 10669911

[95] Rothhammer V Borucki DM Tjon EC Takenaka MC Chao C-C Ardura-Fabregat A . Microglial control of astrocytes in response to microbial metabolites. Nature. (2018) 557:724–8. 10.1038/s41586-018-0119-x 29769726 PMC6422159

[96] Yilmaz C Gökmen V. Determination of tryptophan derivatives in kynurenine pathway in fermented foods using liquid chromatography tandem mass spectrometry. Food Chem. (2018) 243:420–7. 10.1016/j.foodchem.2017.10.004 29146359

[97] Morris MC. Dietary intake of antioxidant nutrients and the risk of incident alzheimer's disease in a biracial community study. JAMA. (2002) 287:3230. 10.1001/jama.287.24.3230 12076219

[98] Engelhart MJ. Dietary intake of antioxidants and risk of Alzheimer's disease. JAMA. (2002) 287:3223. 10.1001/jama.287.24.3223 12076218

[99] Grodstein F Chen J Willett WC. High-dose antioxidant supplements and cognitive function in community-dwelling elderly women. Am J Clin Nutr. (2003) 77:975–84. 10.1093/ajcn/77.4.975 12663300

[100] Tillisch. Consumption of fermented milk product with probiotic modulates brain activity. Gastroenterology. (2013) 144:1–15. 10.1053/j.gastro.2013.02.043.Conflicts PMC3839572 23474283

[101] Oak SJ Jha R. The effects of probiotics in lactose intolerance: a systematic review. Crit Rev Food Sci Nutr. (2019) 59:1675–83. 10.1080/10408398.2018.1425977 29425071

[102] Hayden KM Beavers DP Steck SE Hebert JR Tabung FK Shivappa N . The association between an inflammatory diet and global cognitive function and incident dementia in older women: the Women's Health Initiative Memory Study. Alzheimer's and Dementia. (2017) 13:1187–96. 10.1016/j.jalz.2017.04.004 28531379 PMC5909961

[103] Foligné B Parayre S Cheddani R Famelart MH Madec MN Plé C . Immunomodulation properties of multi-species fermented milks. Food Microbiol. (2016) 53:60–9. 10.1016/j.fm.2015.04.002 26611170

[104] Shen J Zuo ZX Mao AP. Effect of probiotics on inducing remission and maintaining therapy in ulcerative colitis, Crohn's disease, and pouchitis: Meta-analysis of randomized controlled trials. Inflamm Bowel Dis. (2014) 20:21–35. 10.1097/01.MIB.0000437495.30052.be 24280877

[105] Cook PE. Fermented foods as biotechnological resources. Food Res Int. (1994) 27:309–16. 10.1016/0963-9969(94)90099-X

[106] Campbell-Platt G. Fermented foods—A world perspective. Food Res Int. (1994) 27:253–7. 10.1016/0963-9969(94)90093-0

[107] Reddy NR Pierson MD. Reduction in antinutritional and toxic components in plant foods by fermentation. Food Res Int. (1994) 27:281–90. 10.1016/0963-9969(94)90096-5

[108] Steinkraus KH. Nutritional significance of fermented foods. Food Res Int. (1994) 27:259–67. 10.1016/0963-9969(94)90094-9

[109] Kim B Hong VM Yang J Hyun H Im JJ Hwang J . Review of fermented foods with beneficial effects on brain and cognitive function. Prev Nutr Food Sci. (2016) 21:297–309. 10.3746/pnf.2016.21.4.297 28078251 PMC5216880

[110] Bo' B Marino P Tucc G Cherubini C Kirkup K. Systematic review on polyphenol intake and health outcomes: is there sufficient evidence to define a health-promoting polyphenol-rich dietary pattern? Nutrients. (2019) 11:1355. 10.3390/nu11061355 31208133 PMC6627994

[111] Brickman AM Khan UA Provenzano FA Yeung L-K Suzuki W Schroeter H . Enhancing dentate gyrus function with dietary flavanols improves cognition in older adults. Nat Neurosci. (2014) 17:1798–803. 10.1038/nn.3850 25344629 PMC4940121

[112] Turner RS Thomas RG Craft S van Dyck CH Mintzer J Reynolds BA . randomized, double-blind, placebo-controlled trial of resveratrol for Alzheimer's disease. Neurology. (2015) 85:1383–91. 10.1212/WNL.0000000000002035 26362286 PMC4626244

[113] Yuan T Ma H Liu W Niesen DB Shah N Crews R . Pomegranate's neuroprotective effects against Alzheimer's disease are mediated by urolithins, its ellagitannin-gut microbial derived metabolites. ACS Chem Neurosci. (2016) 7:26–33. 10.1021/acschemneuro.5b00260 26559394

[114] Arranz S Chiva-Blanch G Valderas-Martínez P Medina-Remón A Lamuela-Raventós RM Estruch R. Wine, beer, alcohol and polyphenols on cardiovascular disease and cancer. Nutrients. (2012) 4:759–81. 10.3390/nu4070759 22852062 PMC3407993

[115] Mountaki C Dafnis I Panagopoulou EA Vasilakopoulou PB Karvelas M Chiou A . Mechanistic insight into the capacity of natural polar phenolic compounds to abolish Alzheimer's disease-associated pathogenic effects of apoE4 forms. Free Radic Biol Med. (2021) 171:284–301. 10.1016/j.freeradbiomed.2021.05.022 34019932

[116] Carito V Ceccanti M Tarani L Ferraguti GN Chaldakov G Fiore M. Neurotrophins'; modulation by olive polyphenols. Curr Med Chem. (2016) 23:3189–97. 10.2174/0929867323666160627104022 27356540

[117] Moosavi F Hosseini R Saso L Firuzi O. Modulation of neurotrophic signaling pathways by polyphenols. Drug Des Devel Ther. (2015) 23:6936. 10.2147/DDDT.S96936 26730179 PMC4694682

[118] Omar SH. Mediterranean and MIND diets containing olive biophenols reduces the prevalence of Alzheimer's disease. Int J Mol Sci. (2019) 20:2797. 10.3390/ijms20112797 31181669 PMC6600544

[119] Pei R Liu X Bolling B. Flavonoids and gut health. Curr Opin Biotechnol. (2020) 61:153–9. 10.1016/j.copbio.2019.12.018 31954357

[120] Leblhuber F Ehrlich D Steiner K Geisler S Fuchs D Lanser L . The immunopathogenesis of Alzheimer's disease is related to the composition of gut microbiota. Nutrients. (2021) 13:1–34. 10.3390/nu13020361 33504065 PMC7912578

[121] Bravo L. Polyphenols: chemistry, dietary sources, metabolism, and nutritional significance. Nutr Rev. (2009) 56:317–33. 10.1111/j.1753-4887.1998.tb01670.x 9838798

[122] Mrvcic J Posavec S Kazazic S Stanzer D Peša A Stehlik-tomas V. Spirit drinks: a source of dietary polyphenols. Croatian J Food Sci Technol. (2012) 4:102–111.

[123] Thomasson HR. “Gender Differences in Alcohol Metabolism.,” *Recent Developments in Alcoholism*. Boston, MA: Springer US. (2002). p. 163–179 10.1007/0-306-47138-8_9 7624539

[124] Ludwig IA Clifford MN Lean MEJ Ashihara H Crozier A. Coffee: biochemistry and potential impact on health. Food Funct. (2014) 5:1695–717. 10.1039/C4FO00042K 24671262

[125] Shukitt-Hale B Miller MG Chu YF Lyle BJ Joseph JA. Coffee, but not caffeine, has positive effects on cognition and psychomotor behavior in aging. Age (Omaha). (2013) 35:2183–92. 10.1007/s11357-012-9509-4 23344884 PMC3824984

[126] Vauzour D. Dietary polyphenols as modulators of brain functions: biological actions and molecular mechanisms underpinning their beneficial effects. Oxid Med Cell Longev. (2012) 2012:1–16. 10.1155/2012/914273 22701758 PMC3372091

[127] Conte A Pellegrini S Tagliazucchi D. Synergistic protection of PC12 cells from β-amyloid toxicity by resveratrol and catechin. Brain Res Bull. (2003) 62:29–38. 10.1016/j.brainresbull.2003.08.001 14596889

[128] Gleason CE Carlsson CM Barnet JH Meade SA Setchell KDR Atwood CS . preliminary study of the safety, feasibility and cognitive efficacy of soy isoflavone supplements in older men and women. Age Ageing. (2009) 38:86–93. 10.1093/ageing/afn227 19054783 PMC2720778

[129] Mandel SA Winreb O Amit T Youdim MB. Eve Topf center for neurodegenerative diseases research and department of molecular pharmacology, faculty of medicine, Technion, Haifa, Israel. Front Biosci. (2012) 4:581–98. 10.2741/S286 22202078

[130] Socci V Tempesta D Desideri G de Gennaro L Ferrara M. Enhancing human cognition with cocoa flavonoids. Front Nutr. (2017) 4:19. 10.3389/fnut.2017.00019 28560212 PMC5432604

[131] Cimini A Gentile R D'Angelo B Benedetti E Cristiano L Avantaggiati ML . Cocoa powder triggers neuroprotective and preventive effects in a human Alzheimer's disease model by modulating BDNF signaling pathway. J Cell Biochem. (2013) 114:2209–20. 10.1002/jcb.24548 23554028 PMC4170833

[132] Wang J Varghese M Ono K Yamada M Levine S Tzavaras N . Cocoa extracts reduce oligomerization of amyloid-β: implications for cognitive improvement in Alzheimer's disease. J Alzheimer's Dis. (2014) 41:643–50. 10.3233/JAD-132231 24957018

[133] Nehlig A. The neuroprotective effects of cocoa flavanol and its influence on cognitive performance. Br J Clin Pharmacol. (2013) 75:716–27. 10.1111/j.1365-2125.2012.04378.x 22775434 PMC3575938

[134] Sokolov AN Pavlova MA Klosterhalfen S Enck P. Chocolate and the brain: Neurobiological impact of cocoa flavanols on cognition and behavior. Neurosci Biobehav Rev. (2013) 37:2445–53. 10.1016/j.neubiorev.2013.06.013 23810791

[135] CarmanAJ DacksPA LaneRF ShinemanDW FillitHM. Current evidence for the use of coffee and caffeine to prevent age-related cognitive decline and Alzheimer's disease. J Nutr Health Aging. (2014) 18:383–92. 10.1007/s12603-014-0021-724676319

[136] Ozawa M Ninomiya T Ohara T Doi Y Uchida K Shirota T . Dietary patterns and risk of dementia in an elderly Japanese population: the Hisayama study. Am J Clin Nutr. (2013) 97:1076–82. 10.3945/ajcn.112.045575 23553168

[137] Abdolmaleky HM Sheng Y Zhou J-R. Bioactive nutraceuticals oligo-lactic acid and fermented soy extract alleviate cognitive decline in mice in part via anti-neuroinflammation and modulation of gut microbiota. Front Nutr. (2023) 10:6278. 10.3389/fnut.2023.1116278 36969810 PMC10034322

[138] Ko JW Chung Y-S Kwak CS Kwon YH. Doenjang, A Korean traditional fermented soybean paste, ameliorates neuroinflammation and neurodegeneration in mice fed a high-fat diet. Nutrients. (2019) 11:1702. 10.3390/nu11081702 31344808 PMC6723205

[139] Szczerba E Koch M Schlesinger S. Soy consumption, cognitive function, and dementia. Curr Opin Lipidol. (2022) 33:68–75. 10.1097/MOL.0000000000000807 34879041

[140] Rizzo G Baroni L. Soy, soy foods and their role in vegetarian diets. Nutrients. (2018) 10:43. 10.3390/nu10010043 29304010 PMC5793271

[141] Messina M Mejia SB Cassidy A Duncan A Kurzer M Nagato C . Neither soyfoods nor isoflavones warrant classification as endocrine disruptors: a technical review of the observational and clinical data. Crit Rev Food Sci Nutr. (2022) 62:5824–85. 10.1080/10408398.2021.1895054 33775173

[142] Dias GP Cavegn N Nix A do Nascimento Bevilaqua MC Stangl D Zainuddin MSA . The role of dietary polyphenols on adult hippocampal neurogenesis: molecular mechanisms and behavioural effects on depression and anxiety. Oxid Med Cell Longev. (2012) 2012:1–18. 10.1155/2012/541971 22829957 PMC3395274

[143] do Prado FG Pagnoncelli MGB de Melo Pereira GV Karp SG Soccol CR. Fermented soy products and their potential health benefits: a review. Microorganisms. (2022) 10:1606. 10.3390/microorganisms10081606 36014024 PMC9416513

[144] Cheng PF Chen JJ Zhou XY Ren YF Huang W Zhou JJ . Do soy isoflavones improve cognitive function in postmenopausal women? A meta-analysis. Menopause. (2015) 22:198–206. 10.1097/GME.0000000000000290 25003621

[145] Muñoz-Garach A Cornejo-Pareja I Martínez-González MÁ Bulló M Corella D Castañer O . Milk and dairy products intake is related to cognitive impairment at baseline in predimed plus trial. Mol Nutr Food Res. (2021) 65:2000728. 10.1002/mnfr.202000728 33471961

[146] Ozawa M Ohara T Ninomiya T Hata J Yoshida D Mukai N . Milk and dairy consumption and risk of dementia in an elderly Japanese population: the Hisayama study. J Am Geriatr Soc. (2014) 62:1224–30. 10.1111/jgs.12887 24916840

[147] Ogata S Tanaka H Omura K Honda C Hayakawa K. Association between intake of dairy products and short-term memory with and without adjustment for genetic and family environmental factors: a twin study. Clin Nutr. (2016) 35:507–13. 10.1016/j.clnu.2015.03.023 25921203

[148] Bonfili L Cecarini V Cuccioloni M Angeletti M Berardi S Scarpona S . SLAB51 PRobiotic formulation activates SIRT1 pathway promoting antioxidant and neuroprotective effects in an AD mouse model. Mol Neurobiol. (2018) 55:7987–8000. 10.1007/s12035-018-0973-4 29492848 PMC6132798

[149] Mehrabadi S Sadr SS. Assessment of probiotics mixture on memory function, inflammation markers, and oxidative stress in an Alzheimer's disease model of rats. Iran Biomed J. (2020) 24:220–8. 10.29252/ibj.24.4.220 32306720 PMC7275815

[150] Athari Nik Azm S Djazayeri A Safa M Azami K Ahmadvand B Sabbaghziarani F . Lactobacilli and bifidobacteria ameliorate memory and learning deficits and oxidative stress in β-amyloid. (1–42) injected rats. Appl Physiol Nutri and Metabol. (2018) 43:718–26. 10.1139/apnm-2017-0648 29462572

[151] Rezaeiasl Z Salami M Sepehri G. The effects of probiotic lactobacillus and bifidobacterium strains on memory and learning behavior, long-term potentiation. (LTP), and some biochemical parameters in β-amyloid-induced rat's model of Alzheimer's disease. Prev Nutr Food Sci. (2019) 24:265–73. 10.3746/pnf.2019.24.3.265 31608251 PMC6779093

[152] Tomaskova H Kuhnova J Cimler R Dolezal O Kuca K. Prediction of population with Alzheimer's disease in the European Union using a system dynamics model. Neuropsychiatr Dis Treat. (2016) 12:1589–98. 10.2147/NDT.S107969 27418826 PMC4935104

[153] Prince MJ Wu F Guo Y Gutierrez Robledo LM O'Donnell M Sullivan R . The burden of disease in older people and implications for health policy and practice. Lancet. (2015) 385:549–62. 10.1016/S0140-6736(14)61347-7 25468153

[154] Kovač B Raspor P. The use of the mould Rhizopus oligosporus in food production. Food Technol Biotechnol. (1997) 35:69–73.

[155] Battcock M Azam-Ali S. “Fermented Fruits and Vegetables: A Global Perspective.,” *Food & Agriculture Org*. (1998). Available online at: https://www.fao.org/3/x0560e/x0560e00.htm (accessed September 12, 2022).

